# Genome-wide circulating microRNA expression profiling indicates biomarkers for epilepsy

**DOI:** 10.1038/srep09522

**Published:** 2015-03-31

**Authors:** Jun Wang, Jin-Tai Yu, Lin Tan, Yan Tian, Jing Ma, Chen-Chen Tan, Hui-Fu Wang, Ying Liu, Meng-Shan Tan, Teng Jiang, Lan Tan

**Affiliations:** 1 Department of Neurology, Qingdao Municipal Hospital, School of Medicine, Qingdao University, Qingdao, China; 2 Department of Neurology, Qingdao Municipal Hospital, College of Medicine and Pharmaceutics, Ocean University of China, Qingdao, China; 3 Department of Neurology, Qingdao Municipal Hospital, Nanjing Medical University, Qingdao, China; 4 Department of Neurology, Qingdao Municipal Hospital, Dalian Medical University, China; 5 Department of Neurology, Nanjing First Hospital, Nanjing Medical University, Qingdao, China

## Abstract

MicroRNAs (miRNAs) have been proposed as biomarkers for cancer and other diseases due to their stability in serum. In epilepsy, miRNAs have almost been studied in brain tissues and in animals' circulation, but not in circulation of human. To date, a major challenge is to develop biomarkers to improve the current diagnosis of epilepsy. The aim of this study was to evaluate whether circulating miRNAs can be used as biomarkers for epilepsy. We measured the differences in serum miRNA levels between 30 epilepsy patients and 30 healthy controls in discovery and training phases using Illumina HiSeq2000 sequencing followed by quantitative reverse transcriptase polymerase chain reaction (qRT-PCR) assays. The selected miRNAs were then validated in 117 epilepsy patients and 112 healthy controls by qRT-PCR. Let-7d-5p, miR-106b-5p, -130a-3p and -146a-5p were found up-regulated, whereas miR-15a-5p and -194-5p were down-regulated in epilepsy patients compared to controls (P < 0.0001). Among these miRNAs, miR-106b-5p had the best diagnostic value for epilepsy with 80.3% sensitivity and 81.2% specificity. Circulating miRNAs were differentially regulated in epilepsy patients as compared with controls. MiR-106b-5p may serve as a novel, noninvasive biomarker to improve the current diagnosis of epilepsy.

Epilepsy is a chronic neurological disorder characterized by recurring seizures which result from abnormal and synchronous firing of neurons in the brain^1^. It affects approximately 50 million individuals all over the world, representing approximately 4–10‰ of the worldwide population^2^. Although scalp electroencephalograms (EEG) and neuroimaging have been used in clinical practice, the diagnosis of epilepsy is primarily based on a detailed examination of manifestations and a detailed medical history. However, often clinical manifestations and history provided by patients or their families are not sufficient. Therefore, biomarkers may serve as novel diagnostic tools, contributing to the accurate clinical diagnosis and facilitating the appropriate treatment.

MicroRNAs (miRNAs) are single-stranded, small non-coding RNA molecules that regulate gene expression and protein synthesis and are involved in fundamental biological processes^3^. Recently, miRNAs have gained significant attention and have emerged as novel diagnostic tools for many diseases due to their characteristics of stable in serum^4^, easy detection, economical and noninvasive. Notably, circulating miRNAs have been proposed as biomarkers with great accuracy for aging^5^, cancer^4^, and neurological diseases, such as Alzheimer's disease (AD)^6^, multiple sclerosis (MS)^7^, Parkinson's disease (PD)^8^, et al. Over the past 5 years, several target studies and genome-wide miRNA expression profiling studies have demonstrated that miRNAs were de-regulated in epilepsy^9^,^10^,^11^,^12^,^13^,^14^,^15^, but almost all of the studies were based on samples of human brain tissue or animal models.

To systematically evaluate whether circulating miRNAs can serve as biomarkers for epilepsy, we performed, for the first time, genome-wide miRNA expression profiling study in serum of epilepsy patients, and evaluated the association between biomarkers and clinical parameters. In addition, we performed bioinformatics analysis to predict the mRNA targets of selected miRNAs and to explore the potential pathways of targets, and also validated several target mRNAs in peripheral blood monocyte cells (PBMCs).

## Results

### Characteristics of individuals

A total of 289 participants (including 30 epilepsy patients and 30 healthy controls in discovery and training phase, 117 epilepsy patients and 112 controls in validation phase) were recruited to this study. The epilepsy patients comprised of 63 (53.8%) crytogenic, 35 (29.9%) symptomatic, 19 (16.2%) idiopathic aetiology. Among them, 78 (66.7%) patients presented generalized seizures and 39 (33.3%) partial seizures. All patients were on antiepileptic drugs at the last clinical visit, 46 (39.3%) on monotherapy and 71 (60.7%) on polytherapy. The detailed clinical characteristics of individuals were listed in Table 1. No significant differences of age, gender or Body Mass Index (BMI) were found in discovery and training set, or in large-scale validation set (P > 0.05).

### Distinct circulating miRNA profiling of epilepsy vs controls in discovery phase

**Figure 1:**
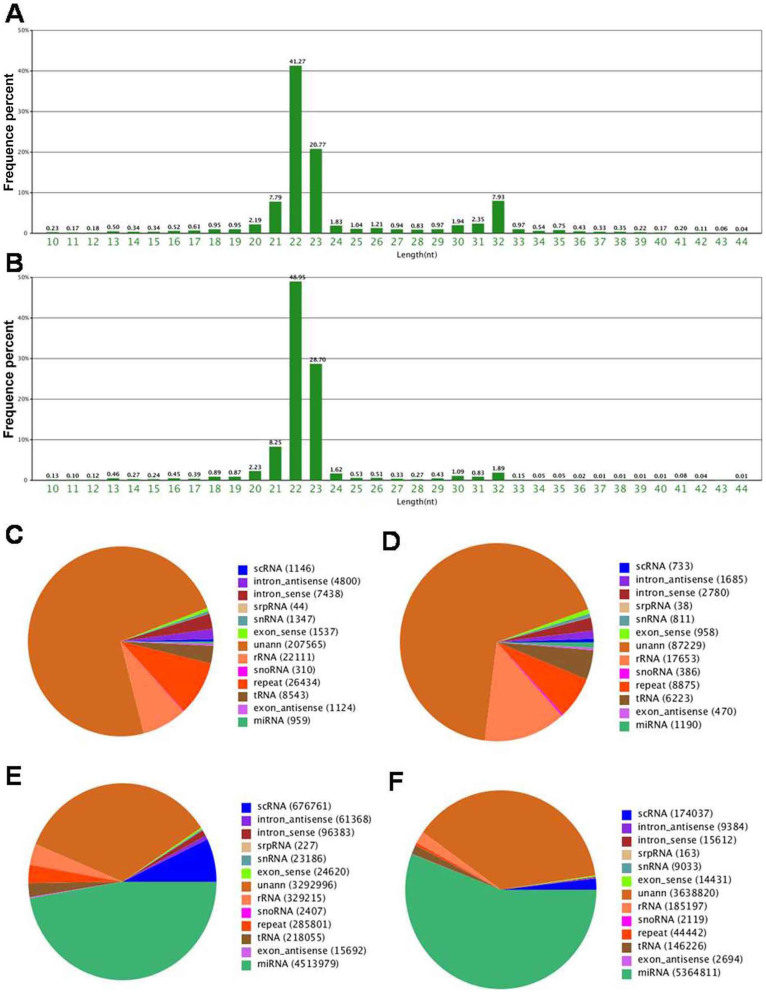
The circulating miRNAs signatures identified by Illumina Hiseq2000 sequencing. The length distribution and frequency percentages of the sequences identified in healthy controls (A) and epilepsy patients (B); RNA species in healthy controls (C) and epilepsy patients (D); and RNA read counts in healthy controls (E) and epilepsy patients (F).

Genome-wide sequencing identified 10,000,000 raw reads in total in both control group and epilepsy group. The dominant small RNAs were 22–23 nt in length, which accounted for 62.04% and 77.65% of the total reads in control and epilepsy groups (Fig. 1A, 1B). After excluding low-quality reads, 3′ adapter-null reads, insert-null reads, 5′ adapter-contaminants reads, reads smaller than 18 nt in length, and reads containing poly A, 9,540,690 (95.72%) clean reads in control group and 9,606,969 (96.40%) clean reads in epilepsy group were remained for further analysis. Among these reads, 6,254,546 (65.56%) reads in control group and 5891828 (61.33%) reads in epilepsy group were perfectly mapped to the human genome in Genbank. Although miRNAs accounted only a tiny fraction of the total small RNAs, the expression levels of individual miRNAs were relatively high. Moreover, both the number of the unique miRNA sequences and the amount of miRNA species were higher in epilepsy patients compared with healthy controls (1190 vs 959, 5364811 vs 4513979, respectively) (Fig. 1C–1F). Among 2,578 serum miRNAs screened by Illumina Hiseq2000 sequencing, 428 miRNAs were detectable in epilepsy patients and 289 miRNAs were detectable in healthy controls. In other serum RNA-Seq studies, the miRNA numbers detected in normal controls ranged from 250 to 380^16^,^17^,^18^. The differences may be attributed to different sequencing technologies, heterogeneity of population, and so on. The deep sequencing data and analyses of differentially expressed miRNAs were listed in Supplementary Table S1. The miRNA levels were considered to be significantly different only if they met the following criteria: (1) having at least 10 copies in epilepsy or control groups; (2) showing a fold-change >2 or <−2 between the two groups (P < 0.05, and P≠0). According to these criteria, we found that 6 miRNAs (miR-144-5p, -15a-5p, -181c-5p, -194-5p, -889-3p and novel-mir-96) were down-regulated and 4 (let-7d-5p, miR-106b-5p, -130a-3p, and -146a-5p) were up-regulated in epilepsy patients compared to controls (Supplementary Table S1). Among the 10 dysregualted miRNAs, novel-mir-96 (sequence: CGTGTGGTTTGGCTGTTCTC) was not listed in miRBase (Release 21, http://www.mirbase.org/) and seems to be novel miRNA.

### Investigation of 10 selected single miRNAs using qRT-PCR in training phase

**Figure 2:**
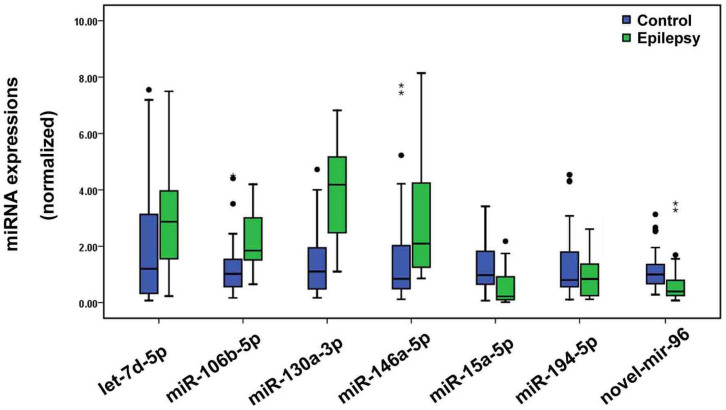
Differential expression levels of significant miRNAs in training phase. Expression levels of the miRNAs were normalized to spiked-in cel-miR-39 and were calculated utilizing the 2^−ΔΔCt^ method. Mann-Whitney U test was used to determine statistical significance. The black dots and stars represent the outliers. The black dots: Values > Q_u_ + 1.5IQR; the stars: Values > Q_u_ + 3.0IQR. Q_u_, upper quartile; IQR, inter-quartile range.

The expression levels of the 10 miRNAs selected by high-throughput sequencing were determined using real-time quantitative reverse transcriptase polymerase chain reaction (qRT-PCR) in a cohort of 30 epilepsy patients and 30 healthy individuals. MiRNA levels were normalized to spiked-in cel-miR-39. All samples were measured in triplicates and the mean values were used for analysis. Only miRNAs with a Cq value < 36, a detection rate > 75% in both groups, and a p value < 0.05 were selected for further analyses^6^. As a result, miR-15a-5p, -194-5p and novel-mir-96 were significantly decreased in epilepsy patients when compared with controls (P = 2.5 × 10^−5^, 7.1 × 10^−22^, 3.6 × 10^−4^, respectively); while let-7d-5p, miR-106b-5p, -130a-3p, and -146a-5p were elevated in epilepsy patients (P = 0.001, 6.0 × 10^−5^, 6.3 × 10^−8^, 0.001, respectively) (Fig. 2). The detection rates of miR-889-3p were less than 75%; no significant difference was observed in the levels of miR-144-5p, and -181c-5p between epilepsy patients and controls (P > 0.05).

### Confirmation of 7 identified single miRNAs using qRT-PCR in validation phase

**Figure 3:**
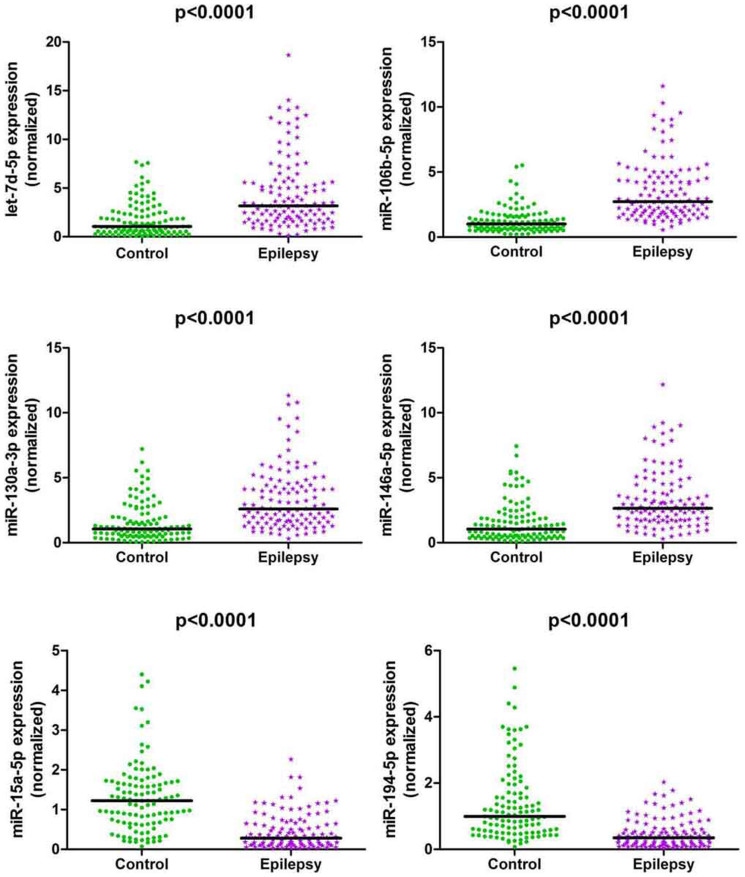
Differential expression levels of significant miRNAs in validation phase. Expression levels of the miRNAs were normalized to spiked-in cel-miR-39 and were calculated utilizing the 2^−ΔΔCt^ method. Mann-Whitney U test was used to determine statistical significance.

**Figure 4:**
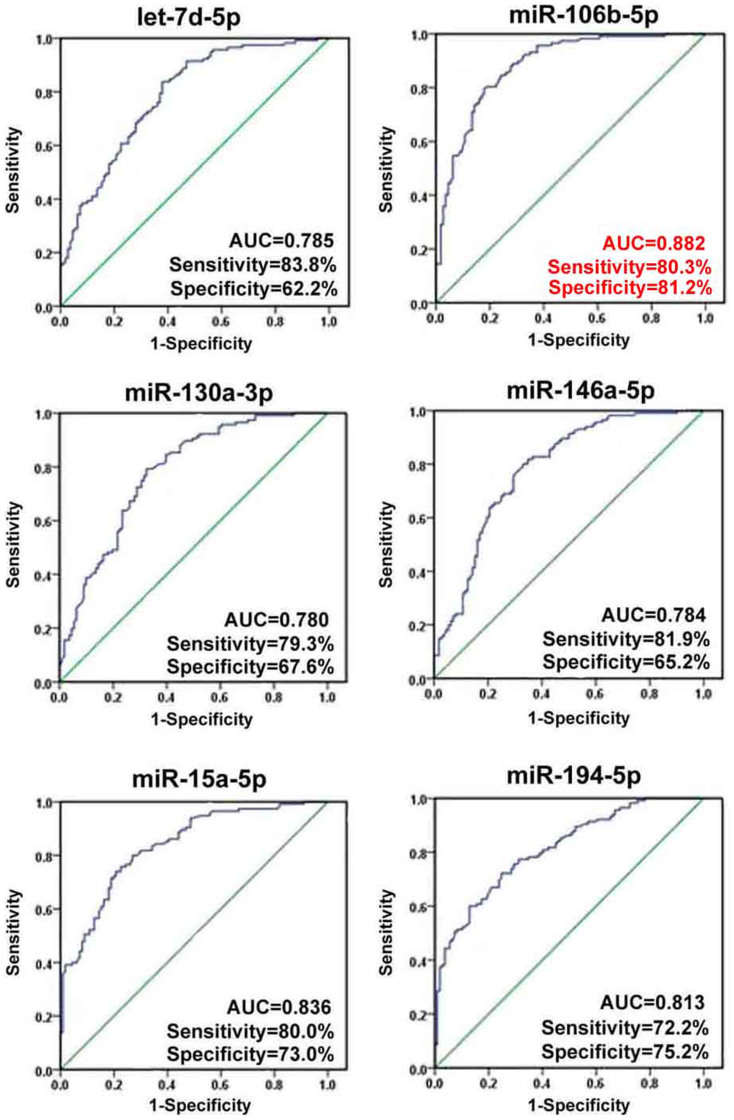
Receiver operating characteristic (ROC) curve analysis using 6 miRNAs selected in validation phase for discriminating epilepsy from healthy controls. For the up-regulated miRNAs, the normalized expression level of miRNAs (2^−ΔΔCt^) was selected as the test variable, and for the down-regulated miRNAs, the logarithm of the normalized expression level (2^−ΔΔCt^) was selected as the test variable. AUC, area under the ROC curve.

To further confirm the expression differences of the 7 miRNAs (let-7d-5p, miR-106b-5p, -130a-3p, -146a-5p, -15a-5p, -194-5p and novel-mir-96) selected in the training phase, the expression levels of these miRNAs were measured on additional 117 epilepsy patients and 112 healthy controls (Supplementary Table S2). The results revealed that let-7d-5p, miR-106b-5p, -130a-3p and -146a-5p were up-regulated, whereas miR-15a-5p and -194-5p were down-regulated in epilepsy patients compared to controls (Fig. 3). Unfortunately, no significant difference was detected in the expression level of novel-mir-96. Receiver Operator Characteristic (ROC) curve analyses indicated that all the 6 significant miRNAs are potential biomarkers for epilepsy diagnosis (Fig. 4). Among these miRNAs, miR-106b-5p showed the highest diagnostic accuracy with an area under the ROC curve (AUC) of 0.882 (95%CI: 0.839–0.926). At the cutoff value of 1.7239 for miR-106b-5p, the optimal sensitivity and specificity were 80.3% and 81.2% respectively. Multivariate logistic regression analyses on variables including age, gender and BMI revealed that miR-106b-5p was a potential biomarker for epilepsy diagnosis (P = 2.11 × 10-11). The odds ratio for cases with expression level of miR-106b-5p more than 1.7239 being associated with epilepsy was 17.710 (95%CI: 9.171–34.199).

In addition to group comparisons, we examined the association between each of the 6 dysregualted miRNAs (let-7d-5p, miR-15a-5p, -194-5p, -146a-5p, -106b-5p and -130a-3p) with clinical parameters. No significant association was found between these 6 miRNAs and age, gender, BMI, disease duration, or seizure frequency (P > 0.05).

Furthermore, we also examined the association between these 6 dysregulated miRNAs with idiopathic generalized epilepsy in 14 patients and 14 matched controls. The results showed that that let-7d-5p, miR-106b-5p, -130a-3p, -15a-5p and -194-5p were significantly up-regulated in patients with idiopathic generalized epilepsy compared with normal controls (P < 0.0001, = 0.035, 0.001, 0.006, 0.008, respectively); while the expression of miR-146a-5p showed no significant difference in two groups (P > 0.05). The discrepancies may be mainly due to the limited sample size with poor representability.

### Bioinformatics analysis

To further understand the biological function of these 6 miRNAs, the target genes were predicted by RNAhybrid and miRanda. We got 361, 48, 8, 19, 87 and 26 intersected targets for let-7d-5p, miR-106b-5p, miR130a-3p, miR-146a-5p, miR-15a-5p and miR-194-5p, respectively (Supplementary Table S3). The subsequent Gene Ontology (GO) analysis was in term of three aspects: molecular function, cellular component and biological process. The GO terms significantly over-represented in deregulated miRNA targets were listed in Supplementary Table S4. The Kyoto Encyclopedia of Genes and Genomes (KEGG) pathway analysis revealed many predicted target genes that were involved in inflammation and neuronal apoptosis, including IRAK1^19^ of miR-146a-5p, CASP6^20^ and MAPKBP1^21^ of miR-106b-5p, MAP2K6^22^ of miR-194-5p, etc. Some of the enriched pathways important in the molecular mechanism of epilepsy were listed in Supplementary Table S5. To functionally validate the role of target genes which have been reported to be involved in inflammation and apoptosis, we quantified the expression levels of some targets mRNAs by RT-PCR from peripheral blood monocyte cells (PBMCs) from 34 epilepsy patients and 32 age-, gender- and BMI-matched normal controls (Supplementary Table S6). We selected GAPDH as internal control. The detailed operation instruction has been described in our previous work^23^. Our results showed that IRAK1 (P = 1.04 × 10^−7^), the targets of miR-146a, MAPKBP1 (P = 10.44 × 10^−7^) and CASP6 (P = 0.001), targets of miR-106b, were down-regulated, whereas MAP2K6 (P = 5.30 × 10^−5^), target of miR-194-5p, was up-regulated in epilepsy patients compared with normal controls. All in all, our findings indicated that these miRNAs may involved in the epileptogenesis through regulating inflammation or apoptosis by inhibiting their related targets.

## Discussion

Up to now, the focus on epilepsy biomarkers for epilepsy diagnosis is mainly based on EEG (e.g. high frequency oscillations) and neuroimaging^24^,^25^,^26^,^27^. However, these biomarkers do not provide high-resolution data sets, and are costly. Therefore, noninvasive, easy detection and economical biomarkers are in need to improve the diagnosis of epilepsy. Recently, miRNAs have been proposed as novel biomarkers for several diseases, including some central nervous system (CNS) diseases^6^,^7^,^8^, due to the characteristics of stable in serum, noninvasive, easy detection and economical. Moreover, the development of powerful detection technologies such as high-throughput sequencing has given a significant boost to the search in miRNAs as biomarkers. Over the past 5 years, several target studies and genome-wide miRNA expression profiling studies have identified changes to over 100 different miRNAs in epilepsy patients and animal models, and provided compelling evidence that epilepsy is associated with widespread changes to miRNA expression.

Here, we provided the first evidence to identify circulating miRNA biomarkers for epilepsy in human. In present study, we first profiled the genome-wide miRNA expression in serum from epilepsy patients. The high-throughput sequencing revealed that miRNAs were differentially expressed in epilepsy patients compared to healthy controls. After 2-stage validation by qRT-PCR, we identified six miRNAs that were significantly dysregulated in the serum of epilepsy patients, namely, let-7d-5p, miR-15a-5p, -194-5p, -106b-5p, -130a-3p and -146a-5p. Among these miRNAs, miR-106b-5p showed the best diagnostic value for epilepsy with 80.3% sensitivity and 81.2% specificity.

Notably, consistent with our findings, previous target studies and genome-wide miRNA expression profiling studies also showed the up-regulated level of miR-146a-5p^10^,^12^,^15^,^28^. However, in Song's study, miR-146a (previous ID of miR-146a-5p) was down-regulated in lithium-pilocarpine-induced chronic temporal lobe epilepsy (TLE) rats^29^. For let-7d-5p, Song and co-workers showed it was up-regulated in TLE rat models^29^, which is consistent with our results; whereas in Mckiernan's study, let-7d-5p was down-regulated in hippocampal and neocortical resection specimens from pharmaco-resistant TLE patients^13^. For miR-106b-5p and miR-130a-3p, contrary to our findings, previous studies have reported that they were down-regulated in epilepsy patients and animal models^12^,^13^. These discrepancies may be explained by the different standards for the selection of TLE patients or varied criteria for the surgery selection of TLE patients and different standards to screen for significantly dysregulated miRNAs. Additionally, limited sample size, different models and/or brain regions, technical factors, extraneous effects including race, BMI, lifestyle, and other individual characteristics may also influence the profiling of miRNA abundance. These required to be validated in the future. For miR-15a-5p and miR-194-5p, no previous literatures have reported their dysregulation in epilepsy or other neurological diseases. Thus replication studies are required in the future to verify our findings.

Emerging evidences highlight that miR-146a may be involved in epileptogenesis through regulating the inflammatory response. MiR-146a has been identified as a key regulator in a feedback system whereby induction of nuclear factor kappa-B (NF-kB) through a myeloid differentiation factor 88 (MyD88)-dependent pathway may up-regulate the miR-146a, which in turn could down-regulate the levels of two key adapter molecules, IL-1RI-associated protein kinases -1 (IRAK1) and -2, and TNF receptor-associated factor 6 (TRAF6) downstream of TLR and cytokine receptors, reducing the activity of this inflammatory pathway^19^,^30^. In our study, we also found that IRAK1 dysregulated in the opposite direction to miR-146a-5p, and associated with inflammation in the KEGG analysis. Moreover, both miR-146a and IL-1β were demonstrated to be up-regulated in astrocytes in epilepsy models^28^,^31^, and IL-1β represents a major pro-inflammatory cytokine involved in the induction of miR-146a^30^,^32^, thus it is possible that expression of miR-146a in astrocytes may represent an attempt to modulate the inflammatory response triggered by IL-1β^28^. With respect to let-7d-5p, it has been found dysregulated in AD^6^ and MS^33^; and in MS, let-7d-5p showed a positive correlation with the pro-inflammatory cytokine IL-1β^33^. AD, MS and epilepsy are all belong to neural diseases, thus it is possible that let-7d-5p may participate the pathogenesis of these three diseases in some common pathways. For miR-106b-5p, -130a-3p, -15a-5p and -194-5p, it has been indicated that these miRNAs were involved in inflammation, apoptosis and cell proliferation in cancers^34^,^35^,^36^,^37^,^38^. However, the mechanisms of these miRNAs in epilepsy or other neural diseases have not been reported. To learn more about the roles of these miRNAs in epilepsy, we predicted the target mRNAs of these miRNAs, performed KEGG pathway analysis, and measured the expression levels of several target mRNAs. Our results indicated that these miRNAs may play a role in epileptogenesis through regulating inflammation or apoptosis by inhibiting their related targets. These still need to be confirmed in the future. In addition, it still remains unclear where these analyzed miRNAs were produced, in which cells and tissues they performed their action and how they function in epileptogensis. In Soreq's research on Parkinson's disease leukocyte miRNAs^39^, they conducted a cell lineage analysis of the exon microarray data using LineageProfiler program to identify the likely cell types and tissues represented in the samples analysed by small RNA deep sequencing and microarrays. Due to the incomplete of some data and the limitation of techniques, we are unable to complete this analysis at present. Future studies should try to overcome these difficulties and resolve these problems.

Our study employed a rigorous approach including a high-throughput sequencing of pooled serum samples followed by multiple qRT-PCR validation sets at the individual level. The high-throughput sequencing could detect the genome-wide miRNA expression, but ignored the individual discrepancies. Thus 2-stage of qRT-PCR was following to verify the different expression levels of selected miRNAs at individual level. Compared to other methods of measuring miRNA expression levels, RT-PCR assay is not affected by genomic DNA contamination, and is a sensitive and accurate method for assessing miRNA expression. In order to make the results of qRT-PCR more accurate, we measured all samples in triplicates and used the mean value for analysis. Besides, our sample size is much larger than that of previous studies, which makes our results more reliable. However, several limitations also exist. First, all participants in our study are from a confined geographic area with less heterogenous background, so future studies in other ethnic populations are needed to verify our findings. Besides, circulating microRNA levels are known to vary dramatically according to a number known and still unknown factors which may affect our results. Therefore, in future work, more than one sample should be taken and assay from patients and controls (biological replicates) in order to improve accuracy of results. Moreover, the function role of disease-associated miRNAs, which are remarkably stable in serum, remains to be solved in the future—whether they are causative or reflect a response to a pathologic situation as a distinct messengers. In addition, as is known, some AEDs could significantly affect liver enzymes and white blood cell count, all these factors could significantly affect circulating microRNAs and not be related to the epilepsy at all. Although we have excluded patients with abnormal blood routine examination and abnormal biochemical examination, whether AEDs could directly affect the miRNA levels remains to be solved in the future.

In summary, we performed a comprehensive investigation of circulating miRNAs in epilepsy and provided the bases to develop circulating miRNAs as accessible biomarkers in epilepsy. In this report, we identified six miRNA biomarkers in epilepsy patients. These results will require further validation in larger-scale prospective studies in different ethnic populations. Moreover, given that a single biomarker is not sufficient for clinical purposes, we do not advocate using miRNA alone for epilepsy diagnosis, but rather combine the findings of the current study with EEG or neuroimaging to improve the current diagnosis of epilepsy. Finally, the miRNAs we have identified have the potential to identify important pathways in epilepsy and thus new targets of therapy.

## Methods

### Study design and patients

**Figure 5:**
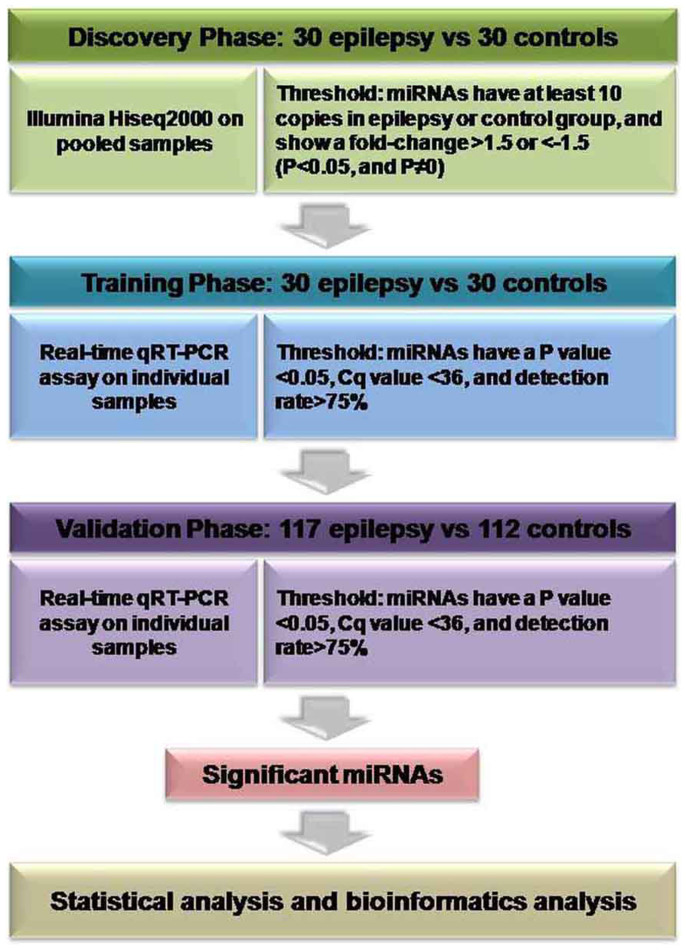
Overview of the study design. qRT-PCR, quantitative reverse transcriptase polymerase chain reaction.

The present study enrolled 147 clinically diagnosed epilepsy patients and 142 healthy controls matched for age, gender and BMI between October, 2013 and June, 2014. A multiphase case-control study was designed to evaluate serum miRNA expression profiling of epilepsy patients and controls (Fig. 5). In the discovery phase, we subjected pooled serum samples from 30 epilepsy patients and 30 controls to Illumina HiSeq 2000 technology to select miRNAs whose expression was altered in epilepsy patients compared to controls. Subsequently, we refined the number of serum miRNAs included as the epilepsy signature by a 2-stage experimental procedure using real-time qRT-PCR assays. The training phase used serum samples from the 30 epilepsy patients and 30 normal controls that had been assessed by Illumina HiSeq 2000 technology, whereas the validation phase used serum samples from additional 117 epilepsy patients and 112 healthy controls.

All epilepsy patients were recruited from the Department of Neurology at Qingdao Municipal Hospital, and several other hospitals in Shandong Province. The patients were diagnosed and classified according to guidelines from the International League against Epilepsy in 2001^40^. All patients were on antiepileptic drugs and went through comprehensive clinical examination, including a medical history, physical and psychiatric examination, laboratory examination, cranial magnetic resonance imaging scans and electroencephalogram. Major exclusion criteria included patients with abnormal blood routine examination, abnormal biochemical examination, febrile convulsions, history of pseudo seizures, autoimmune diseases, allergic response, immune deficiency disorder, diabetes, heart disease, stroke, malignancy, or a systemic or CNS infection 2 weeks before sample collection. The control subjects were recruited from the Health Examination Center of the Qingdao Municipal Hospital, and confirmed healthy and neurologically normal by medical history, general examinations, laboratory examinations, and have no history of seizures or exposure to AEDs. An informed consent to participate in this study was obtained from each subject, and the study protocol was approved by the Ethics Committee of Qingdao Municipal Hospital. All the experiments described here were in accordance with the guidelines and regulations issued by the Ethics Committee of Qingdao Municipal Hospital.

### Blood processing

Up to 6 ml whole blood was collected from each participant and was processed for serum isolation within 3 hours of collection by centrifugation at 3,000 r.p.m. for 5 min at room temperature, followed by a 5 min centrifugation at 12,000 × g at 4°C^6^. The serum samples were stored at −80°C until use, and were not thawed during the period between their collection and use. The hemolytic samples were excluded based on visual detection due to its easy operation and wide application. The thick, red supernatant after centrifugation was considered to be hemolysed sample and excluded.

### Serum small RNA library construction and sequencing

We mixed 300 μl of each serum sample from 30 epilepsy patients and 30 controls respectively. Total RNA of each mixed serum was isolated using a scaled-up version of the mirVana™ PARIS™ Kit (Ambion, USA) protocol^41^: 9 ml mixed serum of each group was transferred to 50 ml tubes, diluted with an equal volume (9 ml) of mirVana™ PARIS™ Kit 2× Denaturing Solution and incubated on ice for 5 min. Then a volume (18 ml) of Acid-Phenol:Chloroform equal to the total of the serum plus 2× Denaturing Solution was added to each tube. The resulting solution was vortexed for 60 sec and centrifuged for 5 min at 12,000 × g at room temperature (25°C). The centrifugation was repeated three times. After that, the aqueous phase was carefully transferred to a fresh tube, mixed thoroughly with 1.25 volumes of 100% ethanol and passed through a mirVana™ PARIS™ Kit volume in sequential 700 μl aliquots. The volume was washed following the manufacturer's protocol, and RNA was eluted in 100 μl of preheated (95°C) elution solution. The concentration and purity of RNA solution were examined by measuring the absorbance at 260–280 nm using the NanoDrop Lite Spectrophotometer (Thermo, Germany). Next, the 18- to 30-nt small RNAs were fractionated, and then were ligated to a 5′ and a 3′ adaptor sequentially. After that, the 5′-, 3′-ligated small RNA solution was reverse-transcribed to cDNA, followed by PCR with primers complementary to the adaptor sequences. Finally, the two generated libraries were sequenced using the Illumina Cluster Station and Genome Analyze (Illumina Inc, CA, USA) at BGI according to the manufacturer's protocol.

### MiRNA quantification by real-time qRT-PCR

Twenty μl total RNA solution was isolated from 400 μl serum of each sample using the mirVana™ PARIS™ Kit according to the manufacturer's protocol. To allow for the normalization of sample-to-sample variation in RNA isolation, synthetic C. elegans miRNA cel-miR-39, which has been recognized as stable control for normalization in serum^16^,^17^,^18^, was added (25 fmol in a 5 μl total volume) to each denatured sample after combining the serum sample with 2× Denaturing Solution^41^. Then the total RNA was reverse transcribed into cDNA in a final volume of 20 μl using One Step PrimeScript miRNA cDNA Synthesis Kit (Takara, Japan). Quantitative real-time PCR was conducted for each sample using SYBR Premix Ex Taq™ II (Takara, Japan) and CFX96 real-time PCR detection system (Bio-Rad, Germany) in a final 25 μl reaction volume according to the manufacturer's protocol. All miRNA primers were purchased from Takara and Tiangen (Beijing, China). At the end of PCR cycles, melting curve analyses were performed to validate the specific generation of the expected PCR products. Each sample was run in triplicates for analysis.

### Bioinformatics analysis

Significant differentially expressed miRNAs in large-scale validation were analyzed by bioinformatics algorithms. Potential targets of these miRNAs were predicted using the microRNA target prediction databases—RNAhybrid and miRanda. Functional annotation was performed by GO to determine the biological significance of these targets, and an p-value calculated by the Fisher's Exact Test indicated which functions were over-represented in the targets. Moreover, the KEGG pathway database was searched for pathway analyses to identify the enriched pathways of targets.

### Statistical analysis

The expression levels of miRNAs for qRT-PCR were normalized to cel-miR-39^42^, and were calculated utilizing the 2^−ΔΔCt^ method^43^. Expression levels of miRNAs were compared using the Kruskall-Wallis test or the Mann-Whitney U test. ROC curves and AUC were established to evaluate the diagnostic value of serum miRNAs for differentiating epilepsy patients with healthy controls. In ROC analysis, the normalized expression level of miRNAs (2^−ΔΔCt^) was selected as the test variable for the up-regulated miRNAs, and for the down-regulated miRNAs, the logarithm of the normalized expression level (2^−ΔΔCt^) was selected as the test variable. The correlations between the variables were assessed with the Pearson's correlation coefficient for quantitative variables with normal distribution, Spearman's correlation coefficient quantitative variables with skewed distribution. Clinical characteristics were compared using χ^2^ test of independence for qualitative variables, t-test of quantitative variables with normal distribution, the non-parametric Kruskall-Wallis test or the Mann-Whitney U test of quantitative variables with skewed distribution. A p value of less than 0.05 was considered statistically significant. All analyses were performed by SPSS 17.0 software (SPSS, Chicago, IL, USA) or Graphpad Prism (version 5.0; Graphpad software).

## Author Contributions

J.W., Lan T. and J.T.Y. designed, performed experiments, analyzed data, and drafted the first draft. J.W., Lan T., Y.T. and J.T.Y. collected the specimens and clinical data. Lin T., J.M. and M.S.T. performed experiments. C.C.T., Y.L., H.F.W. and T.J. analyzed data and drafted the first draft. Lan T. and J.T.Y. designed and supervised experiments. The contents of this study are solely the responsibility of the authors and do not necessarily represent the official view of their institutions or any other party. Lan T. and J.T.Y. have full access to all of the data and take full responsibility for the data, the analyses, and interpretation. All authors reviewed and approved the final report.

## Supplementary Material

Supplementary Information Supplementary information

## Figures and Tables

**Table 1:** Clinical characteristics of individuals

	Discovery and training set		Large-scale validation set	
	Epilepsy	Control	Epilepsy	Control
No.	30	30	117	112
Age, mean ± SD (y)	26.4 ± 11.5	29.2 ± 5.6	29.8 ± 10.3	31.8 ± 9.0
Female:Male	15:15	15:15	57:60	59:53
BMI, mean ± SD (kg/m^2^)	22.5 ± 4.2	23.5 ± 3.4	23.0 ± 4.2	23.6 ± 3.3
Duration, median (range) (y)	4.5(1–32)	NA	6(1–39)	NA
Seizure frequency, median(range) (/6 months)	20(3–118)	NA	12(1–167)	NA
Aetiology				
Symptomatic	8(26.7%)	NA	35(29.9%)	NA
Idiopathic	5(16.7%)	NA	19(16.2%)	NA
Crytogenic	17(56.7%)	NA	63(53.8%)	NA
Seizure type				
Partial	12(40.0%)	NA	39(33.3%)	NA
Generalized	18(60.0%)	NA	78(66.7%)	NA
AED therapy at the last clinic visit				
Monotherapy	13(43.3%)	NA	46(39.3%)	NA
Polytherapy	17(56.7%)	NA	71(60.7%)	NA

Abbreviations: SD, standard deviation; BMI, body mass index; AED, antiepileptic drug; NA, not applicable.
