# Supplementary material for: Genome-wide circulating microRNA expression profiling indicates biomarkers for epilepsy
Source: Sci Rep. 2015 Mar 31;5:9522. doi: 10.1038/srep09522 (PMC4379481; doi:10.1038/srep09522)
Supplement: Supplementary Information [file srep09522-s1.pdf]

Genome-wide circulating microRNA expression profiling indicates biomarkers for epilepsy

Jun Wang, Jin-Tai Yu, Lin Tan, Yan Tian, Jing Ma, Chen-Chen Tan, Hui-Fu Wang, Ying Liu, Meng-Shan Tan, Teng Jiang, Lan Tan

Supplementary Table S1. Differentially expressed miRNAs in epilepsy samples compared to normal subjects by Illumina Hiseq2000 sequencing.

Supplementary Table S2. Mean Cq value of miRNAs in qRT-PCR in the validation phase.

Supplementary Table S3. Target genes of the 6 selected miRNAs.

Supplementary Table S4. GO terms significantly over-represented among the deregulated miRNA targets.

Supplementary Table S5. KEGG pathway analysis results.

Supplementary Table S6. Mean Cq value of mRNAs in RT-PCR.

Supplementary Table S1. Differentially expressed miRNAs in epilepsy samples compared to normal subjects by Illumina Hiseq2000 sequencing.

miR-name	C-expressed	EP-expressed	fold-change(log2 EP/C)	p-value	sig-lable
hsa-let-7d-5p	26	105	2.00380659	1.54E-12	**
hsa-miR-106b-5p	6	83	3.78006198	1.42E-18	**
hsa-miR-130a-3p	167	694	2.04509727	1.97E-76	**
hsa-miR-144-5p	62	14	-2.15680885	1.19E-08	**
hsa-miR-146a-5p	9	123	3.76264396	8.01E-27	**
hsa-miR-15a-5p	80	12	-2.74693901	7.73E-14	**
hsa-miR-181c-5p	28	5	-2.49529212	3.54E-05	**
hsa-miR-194-5p	34	4	-3.09733392	3.01E-07	**
hsa-miR-889-3p	36	9	-2.01001383	3.67E-05	**
novel_mir_96	75	14	-2.4314333	1.43E-11	**
hsa-let-7a-3p	0	10	6.70168766	0.0010144	**
hsa-let-7a-5p	2873	5199	0.84568813	2.29E-47	**
hsa-let-7b-5p	2216	3638	0.70519991	1.03E-75	**
hsa-let-7c-5p	232	392	0.74674066	2.17E-10	**
hsa-let-7d-3p	13	41	1.64709665	0.0001259	**
hsa-let-7e-5p	34	53	0.63045579	0.045608	
hsa-let-7f-5p	3656	12300	1.74033264	0	**
hsa-let-7g-3p	13	24	0.87452687	0.0764263	
hsa-let-7g-5p	610	1427	1.21611587	5.13E-74	**
hsa-let-7i-3p	19	28	0.54944242	0.2017738	
hsa-let-7i-5p	5634	12172	1.10134711	0	**
hsa-miR-100-5p	9147	11197	0.28175397	7.17E-44	**
hsa-miR-101-3p	951	4261	2.15368748	0	**
hsa-miR-103a-3p	5163	11703	1.17060928	0	**
hsa-miR-106b-3p	3667	3607	-0.03378878	0.3179403	
hsa-miR-107	4903	11277	1.19165905	0	**
hsa-miR-10a-5p	99799	106309	0.08117876	2.43E-37	**
hsa-miR-10b-5p	979339	1086134	0.13933414	0	
hsa-miR-1180-3p	12	36	1.57494894	0.0005135	**
hsa-miR-1224-5p	11	6	-0.88461904	0.2320523	
hsa-miR-122-5p	2099	1318	-0.68133956	3.08E-42	**
hsa-miR-1246	41	16	-1.36750871	0.0007845	**
hsa-miR-1255b-5p	18	0	-7.55972123	3.57E-06	**
hsa-miR-125a-3p	18	10	-0.85803357	0.1312061	
hsa-miR-125a-5p	941	654	-0.53489167	2.12E-13	**
hsa-miR-125b-1-3p	17	0	-7.47719159	7.17E-06	**
hsa-miR-125b-2-3p	335	243	-0.47319614	9.07E-05	**
hsa-miR-125b-5p	114	54	-1.08799268	2.50E-06	**
hsa-miR-126-3p	118	231	0.95911304	1.65E-09	**
hsa-miR-126-5p	326	1723	2.39199288	5.69E-69	**
hsa-miR-1270	22	8	-1.46946122	0.0101029	*
hsa-miR-1271-5p	5	10	0.98991746	0.2152331	
hsa-miR-1273h-3p	37	23	-0.69586659	0.0679972	
hsa-miR-1273h-5p	13	9	-0.54054913	0.3954966	
hsa-miR-127-3p	555	333	-0.74695504	3.27E-14	**
hsa-miR-128-1-5p	41	25	-0.72367678	0.0465929	
hsa-miR-128-3p	63	42	-0.59495979	0.0374858	
hsa-miR-1285-3p	28	17	-0.72992043	0.0989077	
hsa-miR-129-5p	28	0	-8.19711839	3.37E-09	**

hsa-miR-1304-3p	25	2	-3.65374508	2.79E-06	**
hsa-miR-1304-5p	11	0	-6.8492487	0.0004684	**
hsa-miR-1307-3p	100	85	-0.24445706	0.2512986	
hsa-miR-1307-5p	491	176	-1.49013848	5.80E-36	**
hsa-miR-130b-3p	83	166	0.9900071	1.58E-07	**
hsa-miR-130b-5p	0	12	6.96474517	0.0002554	**
hsa-miR-134-5p	65	44	-0.57292143	0.0410365	
hsa-miR-139-5p	41	68	0.71991846	0.0107922	
hsa-miR-140-3p	563	941	0.73107184	4.37E-22	**
hsa-miR-141-3p	0	82	9.73733186	2.75E-25	**
hsa-miR-142-3p	0	104	10.08021795	7.09E-32	**
hsa-miR-142-5p	3239	5683	0.80111628	8.13E-92	**
hsa-miR-143-3p	74698	108020	0.52216919	0	**
hsa-miR-144-3p	50	121	1.26501969	5.09E-08	**
hsa-miR-145-3p	41	51	0.30486737	0.3155855	
hsa-miR-145-5p	131	286	1.11645889	2.93E-14	**
hsa-miR-1468-5p	43	18	-1.26635447	0.001182	**
hsa-miR-146b-3p	68	41	-0.73991713	0.0087477	
hsa-miR-146b-5p	3177	3544	0.14772584	2.75E-05	**
hsa-miR-148a-3p	6219	6938	0.14784966	4.29E-09	**
hsa-miR-148a-5p	299	183	-0.71828962	7.49E-08	**
hsa-miR-148b-3p	12	0	-6.97475872	0.0002334	**
hsa-miR-148b-5p	0	10	6.70168766	0.0010144	**
hsa-miR-150-3p	70	27	-1.38436347	8.65E-06	**
hsa-miR-150-5p	12	0	-6.97475872	0.0002334	**
hsa-miR-151a-3p	12462	8829	-0.50720144	5.14E-39	**
hsa-miR-151a-5p	13	0	-7.0902183	0.0001163	**
hsa-miR-151b	30	63	1.06040515	0.0006644	**
hsa-miR-152-3p	0	169	10.78065459	2.40E-51	**
hsa-miR-155-5p	16	0	-7.38973888	1.44E-05	**
hsa-miR-15b-3p	16	8	-1.01001396	0.1040108	
hsa-miR-15b-5p	27	40	0.55702942	0.1209505	
hsa-miR-16-2-3p	188	249	0.39542429	0.0044201	
hsa-miR-16-5p	666	5748	3.09947777	0	**
hsa-miR-17-3p	13	29	1.14751748	0.0146061	*
hsa-miR-17-5p	41	31	-0.41335991	0.2299386	
hsa-miR-181a-2-3p	728	418	-0.81042348	1.09E-20	**
hsa-miR-181a-3p	164	151	-0.12912828	0.4279003	
hsa-miR-181a-5p	11893	15911	0.40992412	9.41E-29	**
hsa-miR-181b-5p	196	289	0.55022586	3.26E-05	**
hsa-miR-181c-3p	79	43	-0.88750523	0.0009469	**
hsa-miR-181d-5p	10	8	-0.33190764	0.636666	
hsa-miR-182-5p	18252	29904	0.70229616	0	
hsa-miR-183-5p	33	0	-8.43416952	1.03E-10	**
hsa-miR-184	9	17	0.90755319	0.1264901	
hsa-miR-185-3p	951	4261	2.15368748	0	**
hsa-miR-185-5p	170	156	-0.13397608	0.4027301	
hsa-miR-186-5p	5879	3866	-0.61471685	3.93E-96	**
hsa-miR-18a-3p	14	0	-7.19711839	5.79E-05	**
hsa-miR-190b	16	12	-0.42499371	0.446961	
hsa-miR-191-5p	19726	19294	-0.04193389	0.0040954	
hsa-miR-192-5p	38582	37561	-0.04868018	3.24E-06	**
hsa-miR-193a-5p	23	19	-0.28562853	0.5273692	
hsa-miR-193b-3p	24	27	0.15998163	0.6962547	

hsa-miR-193b-5p	43	16	-1.4362121	0.0003563	**
hsa-miR-197-3p	45	0	-8.88160345	2.42E-14	**
hsa-miR-199a-3p	239	382	0.66657419	1.41E-08	**
hsa-miR-199a-5p	43	67	0.6298396	0.0245221	
hsa-miR-199b-3p	239	382	0.66657419	1.41E-08	**
hsa-miR-19a-3p	292	110	-1.41845748	1.42E-20	**
hsa-miR-19b-3p	2142	2949	0.45127956	1.46E-28	**
hsa-miR-203a	12	0	-6.97475872	0.0002334	**
hsa-miR-204-5p	37	32	-0.21943796	0.5311871	
hsa-miR-20a-5p	0	25	8.02364348	3.26E-08	**
hsa-miR-20b-3p	10	4	-1.3317344	0.1153298	
hsa-miR-210-3p	94	109	0.2036093	0.3169358	
hsa-miR-2110	0	20	7.70168766	1.03E-06	**
hsa-miR-2115-3p	15	0	-7.29664095	2.89E-05	**
hsa-miR-21-3p	777	1101	0.49283931	2.09E-13	**
hsa-miR-215-5p	538	791	0.54608321	8.36E-12	**
hsa-miR-21-5p	7422	20878	1.47854075	0	**
hsa-miR-221-3p	1191	1313	0.13070603	0.0235288	
hsa-miR-221-5p	59	27	-1.1377135	0.0004653	**
hsa-miR-222-3p	138	154	0.14826795	0.3813512	
hsa-miR-223-3p	316	360	0.17808448	0.1092928	
hsa-miR-223-5p	118	69	-0.78410607	0.0002726	**
hsa-miR-22-3p	31806	20128	-0.67008284	0	**
hsa-miR-224-5p	0	13	7.08023127	0.0001281	**
hsa-miR-2276-3p	12	0	-6.97475872	0.0002334	**
hsa-miR-23a-3p	241	412	0.76362388	3.21E-11	**
hsa-miR-23b-3p	66	85	0.35501002	0.1337242	
hsa-miR-24-3p	1228	946	-0.38638635	5.09E-10	**
hsa-miR-2467-5p	16	0	-7.38973888	1.44E-05	**
hsa-miR-25-3p	17846	28033	0.64153769	0	**
hsa-miR-26a-5p	1434	2669	0.88626674	3.17E-82	**
hsa-miR-26b-3p	17	47	1.45717746	0.0001698	**
hsa-miR-26b-5p	104	261	1.31389869	1.57E-16	**
hsa-miR-27a-3p	210	534	1.33646054	4.67E-33	**
hsa-miR-27a-5p	15	7	-1.10958585	0.089947	
hsa-miR-27b-3p	2944	5069	0.77393562	7.48E-123	**
hsa-miR-28-3p	3816	2351	-0.70877436	1.32E-80	**
hsa-miR-28-5p	0	22	7.83920379	2.58E-07	**
hsa-miR-29a-3p	49	102	1.04772792	1.71E-05	**
hsa-miR-301a-3p	0	19	7.62767979	2.04E-06	**
hsa-miR-30a-3p	255	216	-0.24945083	0.0613413	
hsa-miR-30a-5p	2384	4081	0.76555081	1.83E-97	**
hsa-miR-30b-5p	14	26	0.88311289	0.0626422	
hsa-miR-30c-1-3p	14	7	-1.01006329	0.129629	
hsa-miR-30c-2-3p	52	23	-1.18685223	0.0006858	**
hsa-miR-30c-5p	21	74	1.80713955	3.27E-08	**
hsa-miR-30d-3p	0	25	8.02364348	3.26E-08	**
hsa-miR-30d-5p	1979	2577	0.37093366	5.73E-18	**
hsa-miR-30e-3p	988	769	-0.37151509	7.67E-08	**
hsa-miR-30e-5p	648	364	-0.8456173	6.78E-20	**
hsa-miR-3130-3p	30	0	-8.29664093	8.36E-10	**
hsa-miR-3131	0	11	6.83920379	0.000509	**
hsa-miR-3146	10	0	-6.71163257	0.00094	**
hsa-miR-3150b-3p	26	19	-0.46253851	0.2910944	

hsa-miR-3158-3p	15	10	-0.59495328	0.3182481	
hsa-miR-3177-3p	13	6	-1.12558863	0.1117789	
hsa-miR-3199	14	0	-7.19711839	5.79E-05	**
hsa-miR-3200-3p	8	20	1.31194879	0.0253062	*
hsa-miR-3200-5p	11	7	-0.66219361	0.3513546	
hsa-miR-320a	5556	3581	-0.64367192	1.44E-98	**
hsa-miR-320b	1220	711	-0.78894746	3.92E-32	**
hsa-miR-320c	184	0	-10.91332332	2.15E-56	**
hsa-miR-320d	28	20	-0.49543072	0.2429244	
hsa-miR-323b-3p	0	12	6.96474517	0.0002554	**
hsa-miR-324-3p	14	0	-7.19711839	5.79E-05	**
hsa-miR-324-5p	17	13	-0.39696033	0.4612445	
hsa-miR-32-5p	0	12	6.96474517	0.0002554	**
hsa-miR-331-3p	16	8	-1.01001396	0.1040108	
hsa-miR-335-5p	18	38	1.06799503	0.0081101	**
hsa-miR-338-3p	10	0	-6.71163257	0.00094	**
hsa-miR-338-5p	12	0	-6.97475872	0.0002334	**
hsa-miR-339-3p	42	22	-0.94287709	0.0115558	
hsa-miR-339-5p	50	104	1.04660223	1.44E-05	**
hsa-miR-33b-5p	5	14	1.47538389	0.042949	*
hsa-miR-340-5p	136	73	-0.90761315	9.46E-06	**
hsa-miR-342-3p	280	593	1.07261611	2.86E-26	**
hsa-miR-342-5p	38	14	-1.45052136	0.0007332	**
hsa-miR-345-5p	154	162	0.0630784	0.6983452	
hsa-miR-3605-3p	30	0	-8.29664093	8.36E-10	**
hsa-miR-3605-5p	8	11	0.44946491	0.5134765	
hsa-miR-361-3p	42	36	-0.23237321	0.480498	
hsa-miR-3614-5p	35	0	-8.51904646	2.57E-11	**
hsa-miR-3615	47	64	0.43540755	0.1160436	
hsa-miR-361-5p	44	32	-0.46941791	0.1617484	
hsa-miR-363-3p	474	635	0.41188409	2.30E-06	**
hsa-miR-3648	17	0	-7.47719159	7.17E-06	**
hsa-miR-3675-5p	0	11	6.83920379	0.000509	**
hsa-miR-3688-3p	0	29	8.23773579	2.07E-09	**
hsa-miR-3691-5p	14	0	-7.19711839	5.79E-05	**
hsa-miR-370-3p	8	17	1.07745907	0.0784652	
hsa-miR-371b-5p	0	21	7.77208359	5.15E-07	**
hsa-miR-375	2237	1559	-0.53093223	2.45E-29	**
hsa-miR-377-5p	11	12	0.11549647	0.8522324	
hsa-miR-378a-3p	7422	3360	-1.1533346	0	**
hsa-miR-378c	304	163	-0.90919025	3.10E-11	**
hsa-miR-378d	0	10	6.70168766	0.0010144	**
hsa-miR-381-3p	0	13	7.08023127	0.0001281	**
hsa-miR-3928-3p	10	0	-6.71163257	0.00094	**
hsa-miR-409-3p	597	322	-0.9006597	2.47E-20	**
hsa-miR-409-5p	0	10	6.70168766	0.0010144	**
hsa-miR-410-3p	0	81	9.71962839	5.49E-25	**
hsa-miR-411-5p	0	20	7.70168766	1.03E-06	**
hsa-miR-412-5p	0	24	7.96474517	6.50E-08	**
hsa-miR-421	36	42	0.21240059	0.5197028	
hsa-miR-423-3p	1470	1492	0.01144348	0.8292068	
hsa-miR-423-5p	10051	11826	0.22463549	1.39E-30	**
hsa-miR-424-3p	40	29	-0.4739656	0.1786638	
hsa-miR-424-5p	11	0	-6.8492487	0.0004684	**

hsa-miR-425-3p	0	25	8.02364348	3.26E-08	**
hsa-miR-425-5p	420	401	-0.07677681	0.4461005	
hsa-miR-432-5p	69	54	-0.36363343	0.1656402	
hsa-miR-4433b-5p	11	4	-1.46935054	0.0745335	
hsa-miR-4435	12	0	-6.97475872	0.0002334	**
hsa-miR-4446-3p	10	4	-1.3317344	0.1153298	
hsa-miR-4467	18	9	-1.01005206	0.0838265	
hsa-miR-4504	0	12	6.96474517	0.0002554	**
hsa-miR-450a-5p	18	0	-7.55972123	3.57E-06	**
hsa-miR-450b-5p	0	12	6.96474517	0.0002554	**
hsa-miR-4511	0	11	6.83920379	0.000509	**
hsa-miR-451a	7422	19605	1.39135395	0	**
hsa-miR-452-5p	25	0	-8.03364323	2.72E-08	**
hsa-miR-4526	10	0	-6.71163257	0.00094	**
hsa-miR-454-5p	0	15	7.28669636	3.23E-05	**
hsa-miR-455-5p	15	0	-7.29664095	2.89E-05	**
hsa-miR-4677-3p	81	85	0.05953791	0.7913241	
hsa-miR-4685-3p	184	0	-10.91332332	2.15E-56	**
hsa-miR-4732-3p	36	33	-0.13551671	0.6985725	
hsa-miR-4732-5p	19	26	0.4425196	0.3131642	
hsa-miR-4772-3p	12	0	-6.97475872	0.0002334	**
hsa-miR-483-5p	47	64	0.43540755	0.1160436	
hsa-miR-484	82	102	0.30488096	0.1544174	
hsa-miR-485-5p	11	0	-6.8492487	0.0004684	**
hsa-miR-486-3p	325	250	-0.38849706	0.0013182	**
hsa-miR-486-5p	3037037	3667940	0.26231783	0	**
hsa-miR-490-5p	11	0	-6.8492487	0.0004684	**
hsa-miR-493-3p	10	14	0.47552153	0.4345422	
hsa-miR-493-5p	0	19	7.62767979	2.04E-06	**
hsa-miR-495-3p	16	0	-7.38973888	1.44E-05	**
hsa-miR-500a-3p	305	365	0.249103	0.0258874	
hsa-miR-501-3p	3426	2679	-0.36481801	7.45E-23	**
hsa-miR-501-5p	11	0	-6.8492487	0.0004684	**
hsa-miR-502-3p	46	34	-0.44609952	0.1720703	
hsa-miR-505-3p	11	13	0.23098257	0.7029571	
hsa-miR-5096	18	0	-7.55972123	3.57E-06	**
hsa-miR-532-3p	0	14	7.18715409	6.43E-05	**
hsa-miR-532-5p	1268	1919	0.58781241	5.54E-30	**
hsa-miR-542-3p	12	0	-6.97475872	0.0002334	**
hsa-miR-548e-3p	12	0	-6.97475872	0.0002334	**
hsa-miR-548e-5p	15	9	-0.75051819	0.2204936	
hsa-miR-548k	0	16	7.37981154	1.62E-05	**
hsa-miR-548o-3p	29	27	-0.11305467	0.7712175	
hsa-miR-551b-5p	19	0	-7.63771167	1.78E-06	**
hsa-miR-5683	0	18	7.54966917	4.07E-06	**
hsa-miR-574-3p	23	54	1.22134902	0.0004237	**
hsa-miR-574-5p	280	596	1.07632291	1.69E-26	**
hsa-miR-582-3p	101	87	-0.22525488	0.2864266	
hsa-miR-582-5p	10	0	-6.71163257	0.00094	**
hsa-miR-584-5p	104	123	0.23208366	0.2277099	
hsa-miR-589-5p	90	131	0.53157547	0.0067868	
hsa-miR-598-3p	0	10	6.70168766	0.0010144	**
hsa-miR-616-5p	17	0	-7.47719159	7.17E-06	**
hsa-miR-625-3p	34	9	-1.93110932	9.35E-05	**

hsa-miR-629-5p	98	41	-1.26715835	8.41E-07	**
hsa-miR-6503-3p	11	13	0.23098257	0.7029571	
hsa-miR-6511a-3p	10	0	-6.71163257	0.00094	**
hsa-miR-6514-5p	10	0	-6.71163257	0.00094	**
hsa-miR-652-3p	50	125	1.31194477	1.22E-08	**
hsa-miR-654-3p	0	12	6.96474517	0.0002554	**
hsa-miR-654-5p	0	11	6.83920379	0.000509	**
hsa-miR-660-5p	98	132	0.41969299	0.02869	
hsa-miR-664a-5p	22	9	-1.29951697	0.0190449	*
hsa-miR-671-3p	13	5	-1.38839204	0.0614481	
hsa-miR-6740-5p	16	0	-7.38973888	1.44E-05	**
hsa-miR-675-3p	0	10	6.70168766	0.0010144	**
hsa-miR-6779-5p	0	10	6.70168766	0.0010144	**
hsa-miR-6837-3p	0	17	7.46719795	8.12E-06	**
hsa-miR-6842-3p	0	12	6.96474517	0.0002554	**
hsa-miR-6852-5p	83	31	-1.43084491	6.65E-07	**
hsa-miR-744-5p	142	112	-0.35237847	0.0529291	
hsa-miR-7-5p	0	12	6.96474517	0.0002554	**
hsa-miR-760	12	7	-0.78770364	0.25658	
hsa-miR-7641	15	14	-0.10948685	0.8405606	
hsa-miR-766-3p	7	14	0.99003571	0.1380743	
hsa-miR-769-5p	1112	551	-1.02302005	1.06E-44	**
hsa-miR-7706	141	94	-0.59494435	0.0017984	
hsa-miR-7849-3p	23	12	-0.94856315	0.0622761	
hsa-miR-7976	16	0	-7.38973888	1.44E-05	**
hsa-miR-8061	12	0	-6.97475872	0.0002334	**
hsa-miR-873-3p	136	73	-0.90761315	9.46E-06	**
hsa-miR-873-5p	13	15	0.19647806	0.7251205	
hsa-miR-874-3p	13	9	-0.54054913	0.3954966	
hsa-miR-877-5p	6	14	1.21239536	0.0810783	
hsa-miR-885-5p	10	0	-6.71163257	0.00094	**
hsa-miR-92a-3p	2513	7774	1.61925887	0	**
hsa-miR-92b-3p	36	33	-0.13551671	0.6985725	
hsa-miR-93-5p	210	534	1.33646054	4.67E-33	**
hsa-miR-941	648	318	-1.03695316	2.61E-27	**
hsa-miR-9-5p	0	65	9.40213804	3.40E-20	**
hsa-miR-96-5p	292	110	-1.41845748	1.42E-20	**
hsa-miR-98-5p	30	111	1.87397268	3.68E-12	**
hsa-miR-99a-5p	1104	1187	0.09459159	0.1168583	
hsa-miR-99b-5p	19881	24755	0.30634196	9.25E-111	**
novel_mir_1	13	0	-7.0902183	0.0001163	**
novel_mir_10	15	0	-7.29664095	2.89E-05	**
novel_mir_100	12	0	-6.97475872	0.0002334	**
novel_mir_101	10	0	-6.71163257	0.00094	**
novel_mir_103	15	0	-7.29664095	2.89E-05	**
novel_mir_104	12	0	-6.97475872	0.0002334	**
novel_mir_105	11	0	-6.8492487	0.0004684	**
novel_mir_108	14	0	-7.19711839	5.79E-05	**
novel_mir_109	10	0	-6.71163257	0.00094	**
novel_mir_11	15	0	-7.29664095	2.89E-05	**
novel_mir_110	14	0	-7.19711839	5.79E-05	**
novel_mir_111	10	0	-6.71163257	0.00094	**
novel_mir_112	17	0	-7.47719159	7.17E-06	**
novel_mir_113	10	8	-0.33190764	0.636666	

novel_mir_115	13	0	-7.0902183	0.0001163	**
novel_mir_116	13	0	-7.0902183	0.0001163	**
novel_mir_117	23	0	-7.91330833	1.10E-07	**
novel_mir_118	13	0	-7.0902183	0.0001163	**
novel_mir_12	35	0	-8.51904646	2.57E-11	**
novel_mir_120	11	0	-6.8492487	0.0004684	**
novel_mir_122	12	0	-6.97475872	0.0002334	**
novel_mir_126	11	0	-6.8492487	0.0004684	**
novel_mir_127	33	0	-8.43416952	1.03E-10	**
novel_mir_128	12	0	-6.97475872	0.0002334	**
novel_mir_129	12	0	-6.97475872	0.0002334	**
novel_mir_13	11	0	-6.8492487	0.0004684	**
novel_mir_130	12	0	-6.97475872	0.0002334	**
novel_mir_131	21	0	-7.78208087	4.42E-07	**
novel_mir_133	13	0	-7.0902183	0.0001163	**
novel_mir_134	34	0	-8.4772321	5.16E-11	**
novel_mir_135	13	0	-7.0902183	0.0001163	**
novel_mir_136	15	0	-7.29664095	2.89E-05	**
novel_mir_137	15	0	-7.29664095	2.89E-05	**
novel_mir_138	14	0	-7.19711839	5.79E-05	**
novel_mir_139	17	0	-7.47719159	7.17E-06	**
novel_mir_14	28	0	-8.19711839	3.37E-09	**
novel_mir_140	15	0	-7.29664095	2.89E-05	**
novel_mir_141	12	0	-6.97475872	0.0002334	**
novel_mir_142	11	0	-6.8492487	0.0004684	**
novel_mir_144	12	0	-6.97475872	0.0002334	**
novel_mir_145	27	0	-8.14465824	6.76E-09	**
novel_mir_146	12	0	-6.97475872	0.0002334	**
novel_mir_147	10	0	-6.71163257	0.00094	**
novel_mir_149	11	0	-6.8492487	0.0004684	**
novel_mir_15	20	0	-7.71170138	8.87E-07	**
novel_mir_151	15	0	-7.29664095	2.89E-05	**
novel_mir_152	10	0	-6.71163257	0.00094	**
novel_mir_153	26	0	-8.09021828	1.36E-08	**
novel_mir_155	17	0	-7.47719159	7.17E-06	**
novel_mir_156	15	0	-7.29664095	2.89E-05	**
novel_mir_157	18	0	-7.55972123	3.57E-06	**
novel_mir_158	45	0	-8.88160345	2.42E-14	**
novel_mir_159	40	0	-8.71170138	7.89E-13	**
novel_mir_160	10	0	-6.71163257	0.00094	**
novel_mir_162	25	0	-8.03364323	2.72E-08	**
novel_mir_163	18	0	-7.55972123	3.57E-06	**
novel_mir_164	29	27	-0.11305467	0.7712175	
novel_mir_165	15	0	-7.29664095	2.89E-05	**
novel_mir_166	11	0	-6.8492487	0.0004684	**
novel_mir_167	13	0	-7.0902183	0.0001163	**
novel_mir_168	11	0	-6.8492487	0.0004684	**
novel_mir_169	18	0	-7.55972123	3.57E-06	**
novel_mir_17	11	0	-6.8492487	0.0004684	**
novel_mir_170	14	0	-7.19711839	5.79E-05	**
novel_mir_171	10	0	-6.71163257	0.00094	**
novel_mir_172	10	0	-6.71163257	0.00094	**
novel_mir_173	12	0	-6.97475872	0.0002334	**
novel_mir_174	41	0	-8.74732027	3.93E-13	**

novel_mir_175	12	0	-6.97475872	0.0002334	**
novel_mir_176	11	0	-6.8492487	0.0004684	**
novel_mir_18	11	0	-6.8492487	0.0004684	**
novel_mir_180	13	0	-7.0902183	0.0001163	**
novel_mir_181	20	0	-7.71170138	8.87E-07	**
novel_mir_182	26	0	-8.09021828	1.36E-08	**
novel_mir_183	14	0	-7.19711839	5.79E-05	**
novel_mir_184	15	0	-7.29664095	2.89E-05	**
novel_mir_185	16	0	-7.38973888	1.44E-05	**
novel_mir_186	11	0	-6.8492487	0.0004684	**
novel_mir_187	20	0	-7.71170138	8.87E-07	**
novel_mir_188	16	0	-7.38973888	1.44E-05	**
novel_mir_19	11	0	-6.8492487	0.0004684	**
novel_mir_190	19	0	-7.63771167	1.78E-06	**
novel_mir_191	17	0	-7.47719159	7.17E-06	**
novel_mir_192	18	25	0.46392225	0.3018767	
novel_mir_195	13	0	-7.0902183	0.0001163	**
novel_mir_197	13	0	-7.0902183	0.0001163	**
novel_mir_198	11	0	-6.8492487	0.0004684	**
novel_mir_199	18	0	-7.55972123	3.57E-06	**
novel_mir_2	0	11	6.83920379	0.000509	**
novel_mir_20	11	0	-6.8492487	0.0004684	**
novel_mir_200	14	0	-7.19711839	5.79E-05	**
novel_mir_201	13	0	-7.0902183	0.0001163	**
novel_mir_202	15	0	-7.29664095	2.89E-05	**
novel_mir_206	12	0	-6.97475872	0.0002334	**
novel_mir_207	21	0	-7.78208087	4.42E-07	**
novel_mir_208	15	0	-7.29664095	2.89E-05	**
novel_mir_209	10	0	-6.71163257	0.00094	**
novel_mir_21	12	0	-6.97475872	0.0002334	**
novel_mir_210	38	0	-8.63767544	3.18E-12	**
novel_mir_212	15	0	-7.29664095	2.89E-05	**
novel_mir_213	16	0	-7.38973888	1.44E-05	**
novel_mir_214	13	0	-7.0902183	0.0001163	**
novel_mir_215	12	0	-6.97475872	0.0002334	**
novel_mir_216	17	0	-7.47719159	7.17E-06	**
novel_mir_218	14	0	-7.19711839	5.79E-05	**
novel_mir_219	21	0	-7.78208087	4.42E-07	**
novel_mir_220	12	0	-6.97475872	0.0002334	**
novel_mir_221	12	0	-6.97475872	0.0002334	**
novel_mir_222	18	0	-7.55972123	3.57E-06	**
novel_mir_223	13	9	-0.54054913	0.3954966	
novel_mir_224	17	0	-7.47719159	7.17E-06	**
novel_mir_226	15	0	-7.29664095	2.89E-05	**
novel_mir_227	12	0	-6.97475872	0.0002334	**
novel_mir_229	15	0	-7.29664095	2.89E-05	**
novel_mir_23	14	0	-7.19711839	5.79E-05	**
novel_mir_230	16	0	-7.38973888	1.44E-05	**
novel_mir_231	14	0	-7.19711839	5.79E-05	**
novel_mir_232	17	0	-7.47719159	7.17E-06	**
novel_mir_235	11	0	-6.8492487	0.0004684	**
novel_mir_236	16	0	-7.38973888	1.44E-05	**
novel_mir_238	38	0	-8.63767544	3.18E-12	**
novel_mir_24	11	0	-6.8492487	0.0004684	**

novel_mir_242	12	0	-6.97475872	0.0002334	**
novel_mir_243	14	0	-7.19711839	5.79E-05	**
novel_mir_244	13	0	-7.0902183	0.0001163	**
novel_mir_245	10	0	-6.71163257	0.00094	**
novel_mir_246	10	0	-6.71163257	0.00094	**
novel_mir_247	51	21	-1.2900975	0.0003321	**
novel_mir_248	10	0	-6.71163257	0.00094	**
novel_mir_249	11	0	-6.8492487	0.0004684	**
novel_mir_250	28	20	-0.49543072	0.2429244	
novel_mir_253	21	0	-7.78208087	4.42E-07	**
novel_mir_255	15	0	-7.29664095	2.89E-05	**
novel_mir_256	13	0	-7.0902183	0.0001163	**
novel_mir_258	10	0	-6.71163257	0.00094	**
novel_mir_259	12	0	-6.97475872	0.0002334	**
novel_mir_26	10	0	-6.71163257	0.00094	**
novel_mir_260	14	0	-7.19711839	5.79E-05	**
novel_mir_261	13	0	-7.0902183	0.0001163	**
novel_mir_262	20	0	-7.71170138	8.87E-07	**
novel_mir_263	16	0	-7.38973888	1.44E-05	**
novel_mir_264	15	0	-7.29664095	2.89E-05	**
novel_mir_265	16	0	-7.38973888	1.44E-05	**
novel_mir_266	12	0	-6.97475872	0.0002334	**
novel_mir_267	12	0	-6.97475872	0.0002334	**
novel_mir_268	15	0	-7.29664095	2.89E-05	**
novel_mir_269	14	0	-7.19711839	5.79E-05	**
novel_mir_270	14	0	-7.19711839	5.79E-05	**
novel_mir_271	10	0	-6.71163257	0.00094	**
novel_mir_272	13	0	-7.0902183	0.0001163	**
novel_mir_273	12	0	-6.97475872	0.0002334	**
novel_mir_274	23	0	-7.91330833	1.10E-07	**
novel_mir_275	14	0	-7.19711839	5.79E-05	**
novel_mir_276	12	0	-6.97475872	0.0002334	**
novel_mir_277	13	0	-7.0902183	0.0001163	**
novel_mir_279	34	0	-8.4772321	5.16E-11	**
novel_mir_280	21	0	-7.78208087	4.42E-07	**
novel_mir_281	18	0	-7.55972123	3.57E-06	**
novel_mir_283	18	0	-7.55972123	3.57E-06	**
novel_mir_284	10	0	-6.71163257	0.00094	**
novel_mir_285	19	0	-7.63771167	1.78E-06	**
novel_mir_286	14	0	-7.19711839	5.79E-05	**
novel_mir_287	34	0	-8.4772321	5.16E-11	**
novel_mir_288	11	0	-6.8492487	0.0004684	**
novel_mir_289	18	0	-7.55972123	3.57E-06	**
novel_mir_29	13	0	-7.0902183	0.0001163	**
novel_mir_290	15	0	-7.29664095	2.89E-05	**
novel_mir_291	16	0	-7.38973888	1.44E-05	**
novel_mir_293	10	0	-6.71163257	0.00094	**
novel_mir_294	15	0	-7.29664095	2.89E-05	**
novel_mir_295	16	0	-7.38973888	1.44E-05	**
novel_mir_296	12	0	-6.97475872	0.0002334	**
novel_mir_297	19	0	-7.63771167	1.78E-06	**
novel_mir_298	13	0	-7.0902183	0.0001163	**
novel_mir_299	11	0	-6.8492487	0.0004684	**
novel_mir_3	15	0	-7.29664095	2.89E-05	**

novel_mir_30	292	110	-1.41845748	1.42E-20	**
novel_mir_302	13	0	-7.0902183	0.0001163	**
novel_mir_303	10	0	-6.71163257	0.00094	**
novel_mir_307	13	0	-7.0902183	0.0001163	**
novel_mir_308	13	0	-7.0902183	0.0001163	**
novel_mir_309	13	0	-7.0902183	0.0001163	**
novel_mir_310	11	0	-6.8492487	0.0004684	**
novel_mir_311	22	0	-7.84918614	2.20E-07	**
novel_mir_312	15	0	-7.29664095	2.89E-05	**
novel_mir_313	18	0	-7.55972123	3.57E-06	**
novel_mir_315	12	0	-6.97475872	0.0002334	**
novel_mir_316	19	7	-1.45065658	0.01826	*
novel_mir_317	10	0	-6.71163257	0.00094	**
novel_mir_318	33	0	-8.43416952	1.03E-10	**
novel_mir_319	10	0	-6.71163257	0.00094	**
novel_mir_32	12	0	-6.97475872	0.0002334	**
novel_mir_320	11	0	-6.8492487	0.0004684	**
novel_mir_321	13	0	-7.0902183	0.0001163	**
novel_mir_322	10	0	-6.71163257	0.00094	**
novel_mir_325	176	208	0.23101732	0.1179268	
novel_mir_326	17	0	-7.47719159	7.17E-06	**
novel_mir_328	11	0	-6.8492487	0.0004684	**
novel_mir_329	29	0	-8.24773766	1.68E-09	**
novel_mir_330	15	0	-7.29664095	2.89E-05	**
novel_mir_331	13	0	-7.0902183	0.0001163	**
novel_mir_332	26	0	-8.09021828	1.36E-08	**
novel_mir_333	12	0	-6.97475872	0.0002334	**
novel_mir_334	10	0	-6.71163257	0.00094	**
novel_mir_335	18	0	-7.55972123	3.57E-06	**
novel_mir_337	14	0	-7.19711839	5.79E-05	**
novel_mir_338	18	0	-7.55972123	3.57E-06	**
novel_mir_339	17	0	-7.47719159	7.17E-06	**
novel_mir_34	12	0	-6.97475872	0.0002334	**
novel_mir_340	15	0	-7.29664095	2.89E-05	**
novel_mir_341	13	0	-7.0902183	0.0001163	**
novel_mir_342	14	0	-7.19711839	5.79E-05	**
novel_mir_343	16	0	-7.38973888	1.44E-05	**
novel_mir_344	13	0	-7.0902183	0.0001163	**
novel_mir_345	10	0	-6.71163257	0.00094	**
novel_mir_346	10	0	-6.71163257	0.00094	**
novel_mir_347	26	0	-8.09021828	1.36E-08	**
novel_mir_348	22	0	-7.84918614	2.20E-07	**
novel_mir_349	22	0	-7.84918614	2.20E-07	**
novel_mir_35	12	0	-6.97475872	0.0002334	**
novel_mir_350	11	0	-6.8492487	0.0004684	**
novel_mir_351	17	0	-7.47719159	7.17E-06	**
novel_mir_352	12	0	-6.97475872	0.0002334	**
novel_mir_353	17	0	-7.47719159	7.17E-06	**
novel_mir_354	10	0	-6.71163257	0.00094	**
novel_mir_355	10	0	-6.71163257	0.00094	**
novel_mir_356	18	0	-7.55972123	3.57E-06	**
novel_mir_357	16	0	-7.38973888	1.44E-05	**
novel_mir_359	15	0	-7.29664095	2.89E-05	**
novel_mir_360	10	0	-6.71163257	0.00094	**

novel_mir_361	24	0	-7.97470138	5.47E-08	**
novel_mir_362	18	0	-7.55972123	3.57E-06	**
novel_mir_364	11	0	-6.8492487	0.0004684	**
novel_mir_365	18	0	-7.55972123	3.57E-06	**
novel_mir_366	14	0	-7.19711839	5.79E-05	**
novel_mir_367	17	0	-7.47719159	7.17E-06	**
novel_mir_368	184	0	-10.91332332	2.15E-56	**
novel_mir_369	18	0	-7.55972123	3.57E-06	**
novel_mir_370	22	0	-7.84918614	2.20E-07	**
novel_mir_371	14	0	-7.19711839	5.79E-05	**
novel_mir_374	0	12	6.96474517	0.0002554	**
novel_mir_376	0	10	6.70168766	0.0010144	**
novel_mir_38	11	0	-6.8492487	0.0004684	**
novel_mir_386	0	11	6.83920379	0.000509	**
novel_mir_39	13	0	-7.0902183	0.0001163	**
novel_mir_391	0	12	6.96474517	0.0002554	**
novel_mir_4	42	0	-8.78208087	1.96E-13	**
novel_mir_40	14	0	-7.19711839	5.79E-05	**
novel_mir_400	0	10	6.70168766	0.0010144	**
novel_mir_403	0	10	6.70168766	0.0010144	**
novel_mir_409	0	12	6.96474517	0.0002554	**
novel_mir_41	10	0	-6.71163257	0.00094	**
novel_mir_410	0	10	6.70168766	0.0010144	**
novel_mir_411	0	13	7.08023127	0.0001281	**
novel_mir_414	0	15	7.28669636	3.23E-05	**
novel_mir_418	0	14	7.18715409	6.43E-05	**
novel_mir_42	35	0	-8.51904646	2.57E-11	**
novel_mir_423	0	29	8.23773579	2.07E-09	**
novel_mir_429	0	13	7.08023127	0.0001281	**
novel_mir_43	35	0	-8.51904646	2.57E-11	**
novel_mir_431	0	11	6.83920379	0.000509	**
novel_mir_432	0	14	7.18715409	6.43E-05	**
novel_mir_435	0	11	6.83920379	0.000509	**
novel_mir_44	251	0	-11.36130792	1.15E-76	**
novel_mir_440	0	11	6.83920379	0.000509	**
novel_mir_442	0	13	7.08023127	0.0001281	**
novel_mir_443	0	10	6.70168766	0.0010144	**
novel_mir_449	0	13	7.08023127	0.0001281	**
novel_mir_45	11	0	-6.8492487	0.0004684	**
novel_mir_450	0	10	6.70168766	0.0010144	**
novel_mir_451	0	11	6.83920379	0.000509	**
novel_mir_452	0	169	10.78065459	2.40E-51	**
novel_mir_454	0	11	6.83920379	0.000509	**
novel_mir_457	0	12	6.96474517	0.0002554	**
novel_mir_459	0	14	7.18715409	6.43E-05	**
novel_mir_46	14	0	-7.19711839	5.79E-05	**
novel_mir_460	0	43	8.80603399	1.32E-13	**
novel_mir_461	0	10	6.70168766	0.0010144	**
novel_mir_462	0	11	6.83920379	0.000509	**
novel_mir_464	0	10	6.70168766	0.0010144	**
novel_mir_47	17	0	-7.47719159	7.17E-06	**
novel_mir_470	0	12	6.96474517	0.0002554	**
novel_mir_471	0	11	6.83920379	0.000509	**
novel_mir_478	0	10	6.70168766	0.0010144	**

novel_mir_48	12	0	-6.97475872	0.0002334	**
novel_mir_480	0	10	6.70168766	0.0010144	**
novel_mir_484	0	14	7.18715409	6.43E-05	**
novel_mir_487	0	12	6.96474517	0.0002554	**
novel_mir_488	0	13	7.08023127	0.0001281	**
novel_mir_489	0	20	7.70168766	1.03E-06	**
novel_mir_49	18	0	-7.55972123	3.57E-06	**
novel_mir_490	0	10	6.70168766	0.0010144	**
novel_mir_495	0	13	7.08023127	0.0001281	**
novel_mir_497	0	16	7.37981154	1.62E-05	**
novel_mir_498	0	12	6.96474517	0.0002554	**
novel_mir_50	12	0	-6.97475872	0.0002334	**
novel_mir_501	0	10	6.70168766	0.0010144	**
novel_mir_504	0	14	7.18715409	6.43E-05	**
novel_mir_505	0	33	8.42416629	1.31E-10	**
novel_mir_506	0	12	6.96474517	0.0002554	**
novel_mir_51	42	0	-8.78208087	1.96E-13	**
novel_mir_510	0	10	6.70168766	0.0010144	**
novel_mir_518	0	10	6.70168766	0.0010144	**
novel_mir_519	0	33	8.42416629	1.31E-10	**
novel_mir_52	17	0	-7.47719159	7.17E-06	**
novel_mir_521	0	11	6.83920379	0.000509	**
novel_mir_53	17	0	-7.47719159	7.17E-06	**
novel_mir_530	0	50	9.02364348	1.06E-15	**
novel_mir_533	0	18	7.54966917	4.07E-06	**
novel_mir_55	18	0	-7.55972123	3.57E-06	**
novel_mir_56	42	0	-8.78208087	1.96E-13	**
novel_mir_58	15	0	-7.29664095	2.89E-05	**
novel_mir_59	12	0	-6.97475872	0.0002334	**
novel_mir_60	15	0	-7.29664095	2.89E-05	**
novel_mir_61	14	0	-7.19711839	5.79E-05	**
novel_mir_62	10	0	-6.71163257	0.00094	**
novel_mir_64	16	0	-7.38973888	1.44E-05	**
novel_mir_65	34	0	-8.4772321	5.16E-11	**
novel_mir_66	27	0	-8.14465824	6.76E-09	**
novel_mir_67	136	73	-0.90761315	9.46E-06	
novel_mir_68	15	0	-7.29664095	2.89E-05	**
novel_mir_69	15	0	-7.29664095	2.89E-05	**
novel_mir_7	20	0	-7.71170138	8.87E-07	**
novel_mir_70	27	0	-8.14465824	6.76E-09	**
novel_mir_71	11	0	-6.8492487	0.0004684	**
novel_mir_72	10	0	-6.71163257	0.00094	**
novel_mir_73	15	0	-7.29664095	2.89E-05	**
novel_mir_74	11	0	-6.8492487	0.0004684	**
novel_mir_76	12	0	-6.97475872	0.0002334	**
novel_mir_77	13	0	-7.0902183	0.0001163	**
novel_mir_78	12	0	-6.97475872	0.0002334	**
novel_mir_79	16	0	-7.38973888	1.44E-05	**
novel_mir_80	14	0	-7.19711839	5.79E-05	**
novel_mir_81	19	0	-7.63771167	1.78E-06	**
novel_mir_82	15	0	-7.29664095	2.89E-05	**
novel_mir_83	12	0	-6.97475872	0.0002334	**
novel_mir_84	10	0	-6.71163257	0.00094	**
novel_mir_85	13	0	-7.0902183	0.0001163	**

novel_mir_86	17	0	-7.47719159	7.17E-06	**
novel_mir_88	10	0	-6.71163257	0.00094	**
novel_mir_89	12	0	-6.97475872	0.0002334	**
novel_mir_9	41	31	-0.41335991	0.2299386	
novel_mir_90	11	0	-6.8492487	0.0004684	**
novel_mir_91	13	0	-7.0902183	0.0001163	**
novel_mir_92	30	0	-8.29664093	8.36E-10	**
novel_mir_93	32	0	-8.3897819	2.08E-10	**
novel_mir_94	18	0	-7.55972123	3.57E-06	**
novel_mir_95	12	0	-6.97475872	0.0002334	**
novel_mir_97	10	0	-6.71163257	0.00094	**
novel_mir_98	28	17	-0.72992043	0.0989077	
novel_mir_99	15	0	-7.29664095	2.89E-05	**

Supplementary Table S2. Mean Cq value of miRNAs in qRT-PCR in the validation phase.

Sample	cel-miR-39	let-7d-5p	miR-106b-5p	miR-146a-5p	miR-130a-3p	miR-15a-5p	miR-194-5p
C1	24.96	34.05	27.48	30.61	30.61	28.14	30.54
C2	25.29	32.07	28.57	31.55	31.55	27.27	28.23
C3	25.14	29.83	28.2	29.81	29.81	26.27	29.69
C4	26.08	30.58	28.39	30.79	30.79	30.03	31.76
C5	24.41	28.83	27.21	29.11	29.11	25.96	27.95
C6	25.41	29.51	28.84	28.49	28.49	32.44	31.56
C7	25.09	31.17	27.45	29.09	29.09	27.27	30.1
C8	25.62	32.03	29.29	28.63	28.63	28.13	29.16
C9	24.61	30.75	27	30.89	30.89	28.72	29.47
C10	24.09	29.85	26.56	27.07	27.07	27.17	28.25
C11	25.26	29.75	27.6	32.41	32.41	29.64	28.76
C12	24.24	33.66	28.35	30.91	30.91	26.34	28.84
C13	24.19	32.82	27.85	31.27	31.27	26.75	28.98
C14	24.28	32.02	27.44	28.4	28.4	26.88	27.29
C15	25.06	28.05	28.81	30.5	30.5	27.98	29.57
C16	24.83	30.6	28.72	31.47	31.47	27.5	27.7
C17	24.36	27.75	27.45	31.05	31.05	25.79	29.91
C18	25.13	28.21	29.45	31.68	31.68	26.77	31.79
C19	24.92	30.62	29.46	30.19	30.19	28.23	29.03
C20	24.24	29.87	28.24	29.79	29.79	26.89	28.3
C21	24.24	28.4	29.41	31.25	31.25	25.55	
C22	24.35	32	27.01	29.74	29.74	25.5	29.58
C23	24.46	33.15	28.81	28.95	28.95	26.21	28.43
C24	25.79	29.57	27.78	30.37	30.37	29.63	30.32
C25	24.89	33.05	27.29	30.87	30.87	29.15	30.15
C26	24.01	28.88	27.49	31.21	31.21	25.43	28.24
C27	25.73	33.3	29.35	32.06	32.06	27.87	28.74
C28	25.78	33.54	28.12	31.28	31.28	27.54	30.78
C29	25.62	33.89	28.99	31.6	31.6	28.98	30.46
C30	25.68	33.21	30	30.38	30.38	28.07	28.88
C31	24.49	30.12	27.99	29.6	26.74	26.5	29.75
C32	25.45	30.25	30.14	30.88	30.89	28.8	31.38
C33	25.1	29.74	28.56	31.17	27.34	29.25	29.66
C34	25.12	29.15	28.8	31.26		27.35	30.32
C35	25.97	30.66	29.88	32.62	29.64	29.05	30.09
C36	25.87	30.76	30.27	29.96	29.34	29.7	30.36
C37	25.99	33.48	29.89	32.47	29.11	29.13	
C38	25.98	31.16	30.65	29.98	28.67	31.7	31.59
C39	25.89	33.36	29.97	29.35	28.76	28.72	31.61
C40	25.68	31.2	30.65	28.87	28.24	28.16	28.89
C41	25.35	33.41	28.57	28.05	27.83	26.47	29.9
C42	25.56	32.57	29.84	28.03	27.95	27.91	28.81
C43	25.72	29.81	30.81	28.5	28.15	27.11	31.25
C44	25.96	33.26	30.2	29.29	30.12	31.13	31.6
C45	25.79	31.54	30.81	29.06	29.79	29.06	29.16
C46	25.89	32.85	30.31	29.42	31.58	29.69	28.87
C47	25.52	31.47	28.88	29.55	30.56	28.56	29.38
C48	24.88	31.71	29.11	31.35	29.84	27.38	29.95
C49	25.57		29.76	30.06	30.19	29.57	30.9
C50	25.89	30.26	29.8	30.28	30.58	30.25	29.99

C51	25.52	33.08	29.9	30.07	30.78	30.85	28.81
C52	24.88	33.02	28.37	28.56	29.03	27.75	28.36
C53	25.57	29.95	30.28	30.03	30.24	28.16	30.62
C54	25.99	30.69	30.63	30.15	30.62	29.32	30.91
C55	25.99	34.08	30.11	31.3	30.67	28.76	31.1
C56	25.89	33.65	30.25	31.55	30.45	29.3	29.02
C57	25.89	31.43	30.78	31.29	30.92	28.91	31.1
C58	25.1	33.08	29.62	31.49	28.4	27.25	28.83
C59	25.62	32.92	29.95	29.66	29.61	28.63	28.73
C60	25.97	33.27	30.87	31.05	30.57	29.88	30.99
C61	25.99	33.25	29.8	29.66	31.7	27.71	29.5
C62	25.99	32.64	29.26	30.02	31.09	28.42	28.91
C63	24.28	30.42	27.57	29.49	27.78	28.78	29.88
C64	25.42	32.73	30.13	30.57	30.42	28.21	29.24
C65	25.62	31.05	30.44	30.67	28.96	28.58	31.4
C66	24.08	28.32	27.64	27.81	27.06	26.82	29.49
C67	25.32	30.72	30.16	29.75	27.85	27.53	29.49
C68	24.3	28.94	28.2	29.31	26.64	26.68	28.24
C69	24.11	28.56	28.33	29.61	26.75	26.43	27.8
C70	24.09	29.08	29.12	28.72	26.64	25.19	30.07
C71	24.22	30.11	29.18	29.16	27.85	28.46	29.07
C72	24.13	29.05		29.17	26.82	27.62	27.43
C73	24.03	28.64	28.89	28.14	29.11	26.28	27.85
C74	25.79	31.03	28.96	29.72	28.91	29.23	31.46
C75	24.28	28.9	26.83	27.96	28.75	26.52	28.69
C76	25.82	30.73	29.17	29.31	29.93		30.77
C77	25.65	29.9	30.51	29.97	30.56	29.32	29.02
C78	25.55	34.06	29	30.43	30.49	29.02	31.59
C79	25.67	31.71	29.7	30.18	30.41	28.79	31.33
C80	25.66	33.89	27.96	30.72	30.23	28.35	29.6
C81	25.57	32.83	28.83	30.38	30.71	28.44	29.55
C82	25.36	29.53	27.83	29.87	30.29	27.92	30.05
C83	25.04	29.55	29.44	29.14	27.41	27.52	30.94
C84	25.24	30.11	29.91	30.5	27.92	27.63	28.53
C85	25.4	34.09	27.73	30.07	28.81	27.83	31.41
C86	25.64	31.26	29.51	30.67	28.8	29.8	29.59
C87	24.09	28.81	28.93	29.45	27.1	28.09	
C88	24.93	30.3	29.4	30.91	28.59	29.89	29.87
C89	25.02	29.39	27.5	30.8	29.58	29.64	29.62
C90	24.52	29.81	28.78	30.01	28.37	29.21	29.38
C91	24.07	32.04	27.41	29.41	27.8	25.43	28.96
C92	24.55	31.07	28.24	30.48	28.87	28.7	29.49
C93	24.38	32.41	27.61	30.55	29.59	26.05	29.14
C94	24.29	29.89	28.31	29.56	28.67	28.92	27.48
C95	24.4	28.46	27.47	29.56	29.17	26.45	29.4
C96	24.39	28.32	27.25	30.77	26.45	28.95	29.79
C97	25.08	31.74	29.95	30.22	27.94	27.11	28.04
C98	24.84	31.54	29.08	28.25	30.08	28.14	30.11
C99	25.98	31.74	30.31	32	28.51	29.97	31.01
C100	25.86	29.98	30.69	31.31	31.42	30.46	30.2
C101	25.97	30.85	29.25	32.7	28.27	28.07	32.7
C102	25.98	31.12	30.18	30.66	31.04	29.48	29.45
C103	25.83	31.66	29.95	30.74	29.21	27.83	33.28
C104	25.92	32.71	30.41	31.99	30.76	29.26	30.9

C105	25.76	33.17		29.85	30.68	28.74	30.52
C106	25.56	31.51	30.17	29.58	30.57	28.09	29.87
C107	25.68	32.6	29.4	31.95	30.36	28.02	30.22
C108	24.72	32.87	29.4	30.43	29.76	27.36	28.42
C109	25.07	33.49	29.98	30.12	29.41	27.62	29.53
C110	24.14	31.62	28.23	28.93	28.37	27.77	31.48
C111	25.07	32.77	29.09	29.6	28.42	27.76	28.87
C112	25.06	30.09	28.61	29.47	28.57	27.08	29.12
EP1	25.28	29.74	27.72	28.58	28.58	29.25	31.82
EP2	24.82	29.92	27.23	27.9	27.9	27.39	32.16
EP3	25.15	29.5	26.85	28.11	28.11	29.36	30.96
EP4	25.04	28.46	27.55	27.61	27.61	27.78	31.92
EP5	25.19	30.4	26.15	28.01	28.01	30.64	29.35
EP6	25.29	28.95	28.01	28.97	28.97	30.16	33.49
EP7	24.72	27.4	26.56	27.3	27.3	27.22	28.89
EP8	24.07	28.44	26.08	27.58	27.58	26.81	28.84
EP9	24.14	27.37	26.04	27.1	27.1	25.8	31.21
EP10	24.07	31.96	25.8	27.13	27.13	26.02	30.43
EP11	24.61	27.73	26.72	27.39	27.39	30.16	32.02
EP12	25.71	28.8	26.83	28.11	28.11	28.05	31.78
EP13	24.08	31.25	25.98	26.41	26.41	27.94	30.21
EP14	24.16	28.24	26.17	26.24	26.24	29.34	30.14
EP15	24.08	28.92	26.01	26.81	26.81	28.85	28.3
EP16	25.04	28.76	26.93	27.89	27.89	32.99	31.49
EP17	25.11	28.73	26.52	28.17	28.17	31.32	29.11
EP18	25.08	28.51	26.75	27.85	27.85	33.49	30.55
EP19	24.24	27.25	26.27	26.69	26.69	29.4	30.43
EP20	24.23	28.44	25.99	26.87	26.87	29.35	28.89
EP21	24.34	26.69	25.46	26.31	26.31	30.36	29.42
EP22	24.56	28.63	25.72	26.5	26.5	26.54	27.99
EP23	24.15	28.46	25.81	26.85	26.85	28.9	29.79
EP24	24.22	28.73	25.68	27.02	27.02	25.39	
EP25	24.41	28.17	26.62	27.32	27.32	28.2	31.5
EP26	25.01	28.57	26.49	27.48	27.48	33.14	30.31
EP27	24.87	29.13	26.37	27.89	27.89	33.39	31.44
EP28	24.37	29.84	25.74	27.25	27.25	27.95	32.11
EP29	24.1	30.83	27.19	27.85	27.85	30.92	31.81
EP30	24.11	27.51	25.33	28.08	28.08	28.32	31.24
EP31	24.29	29.01	25.8	27.55	27.55	29.46	29.79
EP32	24.31	29.75	25.61	27.75	27.75	29.73	32
EP33	24.73	29.54	26.04	27.87	27.87	29.98	31.19
EP34	24.84	28.32	26.12	27.92	27.92	30.52	31.52
EP35	25.98	29.13	27.57	28.56	28.56	29.59	32.89
EP36	25.86	28.98	27.89	28.37	28.37	29.32	31.29
EP37	25.97	31.44	27.88	29.1	29.1	29.51	33.87
EP38	25.98	30.48	27.26	28.76	28.76	28.91	33.65
EP39	25.83	30.36	27.84	28.55	28.55	28.85	32.05
EP40	25.92	30.17	28.6	28.04	28.04	29.26	30.2
EP41	25.76	31.32	26.96	28.25	28.25	31.76	30.85
EP42	25.56	30.57	26.69	27.75	27.75	28.71	29.8
EP43	25.68	29.87	27.7	28.2	28.2	29.83	30.7
EP44	24.72	29.83	25.73	26.69	26.69	30.27	30.14
EP45	25.07	28.55	27.23	28.02	28.02	31.19	30.43
EP46	24.14	27.5	25.53	27.14	27.14	30.54	31.37

EP47	25.07	28.19	25.64	29.16	29.16	28.3	30.23
EP48	25.06	28.3	26.99	28.7	28.7	29.02	31.48
EP49	24.95	29.58	26.35	28.7	28.7	29.81	29.51
EP50	24.74	26.93	27.11	28.9	28.9	29.24	29.31
EP51	25.24	27.8	25.97	28.44	28.44	29.63	29.64
EP52	24.06	27.68	24.71	27.54	27.54	28.53	31.16
EP53	25.47	28.53		28.13	28.13	29.74	32.69
EP54	24.26	29.15	25.01	26.86	26.86	27.93	29.69
EP55	24.59	29.53	26.74	27.87	27.87	29.72	30.63
EP56	24.29	29.32	26.57	27.66	27.66	30.28	30.14
EP57	24.15	27.31	24.84	26.84	26.84	30.45	30.81
EP58	25.79	29.36	27.82	28.71	28.71	32.52	31.93
EP59	25.58	29.33	27.61	28.81	28.81	33	32.07
EP60	25.86	29.24	27.99	29.11	29.11	32.96	32.06
EP61	24.93	28.97	26.21	27.4	27.4	31.23	30.3
EP62	24.23	28.14	26.52	27.96	27.96	31.62	31.66
EP63	24.63	27.65	26.9	27.32	27.32	31.44	31.8
EP64	24.66	27.93	26.78	26.81	26.81	31.63	31.37
EP65	24.57	27.68	27.07	27.57	27.57	31.36	31.06
EP66	24.15	28.2	27.01	28.06	28.06	28.17	32.54
EP67	24.89	27.73	27.51	28.93	28.93	29.32	32.89
EP68	25.08	29.63	27.47	29.15	29.15	28.97	31.99
EP69	24.41	29.33	27.39	28.8	28.8	28.76	32.44
EP70	24.01	29.02	24.63	28.93	28.93	28.39	30.51
EP71	25.79	30.47	28.17	28.73	28.73	30.13	32.01
EP72	24.35	29.39	25.51	26.82	26.82	29.38	32.68
EP73	24.35	28.69	25.68	27.7	27.7	27.6	30.95
EP74	24.9	27.13	25.45	27.88	27.88	28.01	32.18
EP75	24.07	29.74	25.6	24.97	26.97	29.42	31.43
EP76	25.01	28.48	26.31	28.51	28.51	28.37	32.97
EP77	24.31	28.27	25.74	27.05	25.82	28.04	30.31
EP78	24.44	30.29	25.82	28.62	25.74	28.77	30.63
EP79	25.56	30.68	26.25	29.17	26.87	29.37	31.99
EP80	24.14	28.41	26.31	27.31	25.42	27.75	30.4
EP81	24.03	26.89	25.69	26.98	25.62	27.28	29.48
EP82	24.84	28.89	28.01	28.08	26.87	30.02	30.97
EP83	25.98	29.1	28.51	29.31	27.89	29.87	32.93
EP84	24.72	29.56	27.96	28.04	26	29.89	30.22
EP85	25.87	30.95	28.99	28.93	28.88	30.18	30.8
EP86	24.11	27.23	26.44	27.28	24.79	27.64	31.86
EP87	24.29	28.76	26.94	30.13	25.49	27.67	29.08
EP88	24.68	28.79	27.07	28.29	25.81	27.68	30.12
EP89	24.94	29.85	27.72	28.84	26.96	30.66	30.49
EP90	25.86	30.8	29.86	30.69	28.87	29.68	32.4
EP91	24.14	28.88	25.67	27.37	26.3	27.96	29.01
EP92	25.47	30.04	26.99	28.96	26.86	29.71	30.71
EP93	24.22	29.36	24.48	27.25	24.79	28.24	28.55
EP94	25.91	30.84	27.61	30.03	27.84	29.4	30.74
EP95	25.72	28.09	28.94	29.89	27.12	29.87	31
EP96	24.27	26.95	28.1	29.11	26.64	30.21	31.22
EP97	25.79	29.2	29.21	30.06	29.52	29.99	31.05
EP98	25.89	29.05	29.36	30.18	29.54	30.85	31.82
EP99	25.78	28.79	27.6	29.67		30.8	30.34
EP100	25.91	29.57	28.43	30.07	28.66	31.11	31.11

EP101	24.92	29.48	27.71	30.26	27.07	28.11	29.04
EP102	25.24	29.09	27.72	30.04	27.52	28.28	29.73
EP103	24.05	27.78	26.47	28.69	24.74	27.73	
EP104	24.1	28.42	26.25	28.52	25.13	28.48	29.71
EP105	25.05	30.26	28.18	29.37	27.08	28.84	30.77
EP106	24.88	29.26	27.76	29.81	27.01	27.74	33.09
EP107	25.19	29.96	28.66	30.56	26.47	28.37	29.87
EP108	24.24	26.3	25.8	27.99	26.53	29.62	29.15
EP109	25.72	28.58	27.72		27.99		31.79
EP110	24.07	29.31	25.36	27.55	26.19	27.97	28.16
EP111	25.47	28	29.22	29.28	27.97	29.64	29.49
EP112	25.73	31.29	28.45	28.95	28.59	29.96	32
EP113	24.32	26.62	27.01	27.15	26.94	30.6	30.03
EP114	25.39	30.45	29.05	29.02	27.78	30.53	30.44
EP115	25.1	28.48	28.16	28.21	27.27		29.89
EP116	25.89	30.73	28.95	29.11	27.55	31.49	30.42
EP117	24.27	27.6	26.28	26.74	27.44	28.92	28.68

Abbreviation: All samples were measured in triplicates,and the Cq values we provided are the mean value. The blank spaces were those Cq values more than 36.

Supplementary Table S3. Target genes of the 6 selected miRNAs.

miRNA_name	target_name	symol
hsa-let-7d-5p	NM_001080442	SLC38A8
hsa-let-7d-5p	NM_001029864	KIAA1755
hsa-let-7d-5p	NM_001102421	ZFAND5
hsa-let-7d-5p	NM_006007	ZFAND5
hsa-let-7d-5p	NM_001102420	ZFAND5
hsa-let-7d-5p	NM_207411	XKR5
hsa-let-7d-5p	NM_207364	GPR148
hsa-let-7d-5p	NM_006195	PBX3
hsa-let-7d-5p	NM_174911	FAM84B
hsa-let-7d-5p	NM_001624	AIM1
hsa-let-7d-5p	NM_001193552	ZNF850
hsa-let-7d-5p	NR_037596	FLJ14186
hsa-let-7d-5p	NM_005128	DOPEY2
hsa-let-7d-5p	NM_001134778	PBX3
hsa-let-7d-5p	NM_052888	LRRC37B
hsa-let-7d-5p	NR_038109	SNHG16
hsa-let-7d-5p	NR_038110	SNHG16
hsa-let-7d-5p	NR_038111	SNHG16
hsa-let-7d-5p	NR_038108	SNHG16
hsa-let-7d-5p	NM_001159293	ZNF737
hsa-let-7d-5p	NR_027299	XPC
hsa-let-7d-5p	NR_024122	PBX3
hsa-let-7d-5p	NR_024123	PBX3
hsa-let-7d-5p	NM_014215	INSRR
hsa-let-7d-5p	NR_038292	LOC100507173
hsa-let-7d-5p	NR_003654	SCAND2
hsa-let-7d-5p	NR_004859	SCAND2
hsa-let-7d-5p	NM_001008397	GPX8
hsa-let-7d-5p	NM_001242740	LOC100507462
hsa-let-7d-5p	NR_040054	IQCH-AS1
hsa-let-7d-5p	NR_040085	TRG-AS1
hsa-let-7d-5p	NM_001242928	ZNF410
hsa-let-7d-5p	NM_018212	ENAH
hsa-let-7d-5p	NM_001008493	ENAH
hsa-let-7d-5p	NM_001242924	ZNF410
hsa-let-7d-5p	NM_005630	SLCO2A1
hsa-let-7d-5p	NM_014668	GREB1
hsa-let-7d-5p	NM_000343	SLC5A1
hsa-let-7d-5p	NR_033411	SLC5A1
hsa-let-7d-5p	NM_001034	RRM2
hsa-let-7d-5p	NM_004091	E2F2
hsa-let-7d-5p	NM_001652	AQP6
hsa-let-7d-5p	NM_001165931	RRM2
hsa-let-7d-5p	NM_153442	GPR26
hsa-let-7d-5p	NM_016124	RHD
hsa-let-7d-5p	NM_001874	CPM
hsa-let-7d-5p	NM_198320	CPM
hsa-let-7d-5p	NM_001005502	CPM
hsa-let-7d-5p	NM_021098	CACNA1H
hsa-let-7d-5p	NM_001005407	CACNA1H

hsa-let-7d-5p	NM_014228	SLC6A7
hsa-let-7d-5p	NM_001173452	TFCP2
hsa-let-7d-5p	NM_001173453	TFCP2
hsa-let-7d-5p	NM_014762	DHCR24
hsa-let-7d-5p	NM_005798	TRIM13
hsa-let-7d-5p	NM_052811	TRIM13
hsa-let-7d-5p	NM_213590	TRIM13
hsa-let-7d-5p	NM_004707	ATG12
hsa-let-7d-5p	NR_033362	ATG12
hsa-let-7d-5p	NR_033363	ATG12
hsa-let-7d-5p	NM_000859	HMGCR
hsa-let-7d-5p	NM_001947	DUSP7
hsa-let-7d-5p	NM_001130996	HMGCR
hsa-let-7d-5p	NM_001008530	LGMN
hsa-let-7d-5p	NM_001395	DUSP9
hsa-let-7d-5p	NM_005787	ALG3
hsa-let-7d-5p	NM_001006941	ALG3
hsa-let-7d-5p	NR_024533	ALG3
hsa-let-7d-5p	NR_024534	ALG3
hsa-let-7d-5p	NM_001163	APBA1
hsa-let-7d-5p	NM_001139	ALOX12B
hsa-let-7d-5p	NM_001382	DPAGT1
hsa-let-7d-5p	NM_014830	ZBTB39
hsa-let-7d-5p	NM_182907	PRDM1
hsa-let-7d-5p	NM_014834	LRRC37A
hsa-let-7d-5p	NM_005610	RBBP4
hsa-let-7d-5p	NM_001135255	RBBP4
hsa-let-7d-5p	NM_001135256	RBBP4
hsa-let-7d-5p	NM_001127691	RHD
hsa-let-7d-5p	NR_015451	LINC00294
hsa-let-7d-5p	NM_001168335	ME2
hsa-let-7d-5p	NM_001168335	ME2
hsa-let-7d-5p	NM_004996	ABCC1
hsa-let-7d-5p	NM_019900	ABCC1
hsa-let-7d-5p	NM_000172	GNAT1
hsa-let-7d-5p	NM_019862	ABCC1
hsa-let-7d-5p	NM_144499	GNAT1
hsa-let-7d-5p	NM_019899	ABCC1
hsa-let-7d-5p	NM_019898	ABCC1
hsa-let-7d-5p	NM_012193	FZD4
hsa-let-7d-5p	NM_003922	HERC1
hsa-let-7d-5p	NM_004391	CYP8B1
hsa-let-7d-5p	NM_014244	ADAMTS2
hsa-let-7d-5p	NM_002396	ME2
hsa-let-7d-5p	NM_002396	ME2
hsa-let-7d-5p	NM_005121	MED13
hsa-let-7d-5p	NM_004101	F2RL2
hsa-let-7d-5p	NM_001198	PRDM1
hsa-let-7d-5p	NM_002268	KPNA4
hsa-let-7d-5p	NM_003417	ZNF264
hsa-let-7d-5p	NM_002881	RALB
hsa-let-7d-5p	NM_004532	MUC4
hsa-let-7d-5p	NM_138297	MUC4
hsa-let-7d-5p	NM_006286	TFDP2

hsa-let-7d-5p	NM_018406	MUC4
hsa-let-7d-5p	NM_001178138	TFDP2
hsa-let-7d-5p	NM_007292	ACOX1
hsa-let-7d-5p	NM_001178139	TFDP2
hsa-let-7d-5p	NM_004035	ACOX1
hsa-let-7d-5p	NM_005374	MPP2
hsa-let-7d-5p	NM_001178140	TFDP2
hsa-let-7d-5p	NM_001185039	ACOX1
hsa-let-7d-5p	NM_001178141	TFDP2
hsa-let-7d-5p	NM_004714	DYRK1B
hsa-let-7d-5p	NM_001178142	TFDP2
hsa-let-7d-5p	NM_002972	SBF1
hsa-let-7d-5p	NM_001007278	TRIM13
hsa-let-7d-5p	NM_001640	APEH
hsa-let-7d-5p	NM_002337	LRPAP1
hsa-let-7d-5p	NM_014712	SETD1A
hsa-let-7d-5p	NM_000821	GGCX
hsa-let-7d-5p	NM_001142269	GGCX
hsa-let-7d-5p	NR_037570	NFS1
hsa-let-7d-5p	NM_005540	INPP5B
hsa-let-7d-5p	NM_005540	INPP5B
hsa-let-7d-5p	NR_027409	GOLGA8A
hsa-let-7d-5p	NM_012264	TMEM184B
hsa-let-7d-5p	NM_007283	MGLL
hsa-let-7d-5p	NM_001195071	TMEM184B
hsa-let-7d-5p	NM_001003794	MGLL
hsa-let-7d-5p	NM_001195072	TMEM184B
hsa-let-7d-5p	NM_018247	TMEM30A
hsa-let-7d-5p	NM_007347	AP4E1
hsa-let-7d-5p	NM_016324	ZNF274
hsa-let-7d-5p	NM_016325	ZNF274
hsa-let-7d-5p	NM_014939	TRAPPC8
hsa-let-7d-5p	NM_018217	EDEM2
hsa-let-7d-5p	NM_001145025	EDEM2
hsa-let-7d-5p	NM_012320	PLA2G15
hsa-let-7d-5p	NM_018390	PLCXD1
hsa-let-7d-5p	NM_018390	PLCXD1
hsa-let-7d-5p	NM_133502	ZNF274
hsa-let-7d-5p	NM_018662	DISC1
hsa-let-7d-5p	NM_001012957	DISC1
hsa-let-7d-5p	NM_015506	MMACHC
hsa-let-7d-5p	NM_015177	DTX4
hsa-let-7d-5p	NM_015409	EP400
hsa-let-7d-5p	NM_014982	PCNX
hsa-let-7d-5p	NM_001195610	DCDC2
hsa-let-7d-5p	NM_020914	RNF213
hsa-let-7d-5p	NM_015490	SEC31B
hsa-let-7d-5p	NM_018129	PNPO
hsa-let-7d-5p	NM_017728	TMEM104
hsa-let-7d-5p	NM_022047	DEF6
hsa-let-7d-5p	NM_014339	IL17RA
hsa-let-7d-5p	NM_183416	KIF1B
hsa-let-7d-5p	NM_017672	TRPM7
hsa-let-7d-5p	NM_006589	FAM189B

hsa-let-7d-5p	NR_026728	EDEM2
hsa-let-7d-5p	NM_198264	FAM189B
hsa-let-7d-5p	NM_001143958	TMEM30A
hsa-let-7d-5p	NM_019018	FAM105A
hsa-let-7d-5p	NM_017964	SLC30A6
hsa-let-7d-5p	NM_001193513	SLC30A6
hsa-let-7d-5p	NM_021180	GRHL3
hsa-let-7d-5p	NM_015029	POP1
hsa-let-7d-5p	NM_001193514	SLC30A6
hsa-let-7d-5p	NM_198173	GRHL3
hsa-let-7d-5p	NM_001145860	POP1
hsa-let-7d-5p	NM_001145861	POP1
hsa-let-7d-5p	NM_001193515	SLC30A6
hsa-let-7d-5p	NM_001195010	GRHL3
hsa-let-7d-5p	NM_014049	ACAD9
hsa-let-7d-5p	NM_177538	CYP20A1
hsa-let-7d-5p	NR_033426	ACAD9
hsa-let-7d-5p	NM_020119	ZC3HAV1
hsa-let-7d-5p	NM_006871	RIPK3
hsa-let-7d-5p	NM_012153	EHF
hsa-let-7d-5p	NM_001206615	EHF
hsa-let-7d-5p	NM_018411	HR
hsa-let-7d-5p	NM_005144	HR
hsa-let-7d-5p	NM_015540	RPAP1
hsa-let-7d-5p	NM_006628	ARPP19
hsa-let-7d-5p	NM_016356	DCDC2
hsa-let-7d-5p	NM_013302	EEF2K
hsa-let-7d-5p	NM_001164537	DISC1
hsa-let-7d-5p	NM_001164540	DISC1
hsa-let-7d-5p	NM_001007561	IRGQ
hsa-let-7d-5p	NM_024674	LIN28A
hsa-let-7d-5p	NR_002328	GNRHR2
hsa-let-7d-5p	NM_144578	MAPK1IP1L
hsa-let-7d-5p	NM_145034	TOR1AIP2
hsa-let-7d-5p	NM_148886	SMCR7
hsa-let-7d-5p	NM_001199260	TOR1AIP2
hsa-let-7d-5p	NM_030784	GPR63
hsa-let-7d-5p	NM_001144900	SMCR7
hsa-let-7d-5p	NM_025188	TRIM45
hsa-let-7d-5p	NM_001145635	TRIM45
hsa-let-7d-5p	NM_001167942	TNFAIP8L1
hsa-let-7d-5p	NM_030640	DUSP16
hsa-let-7d-5p	NM_152362	TNFAIP8L1
hsa-let-7d-5p	NM_032826	SLC35B4
hsa-let-7d-5p	NM_139162	SMCR7
hsa-let-7d-5p	NM_032116	KATNAL1
hsa-let-7d-5p	NM_001014380	KATNAL1
hsa-let-7d-5p	NM_032828	ZNF587
hsa-let-7d-5p	NM_001204817	ZNF587
hsa-let-7d-5p	NM_024341	ZNF557
hsa-let-7d-5p	NM_001044387	ZNF557
hsa-let-7d-5p	NM_033655	CNTNAP3
hsa-let-7d-5p	NM_001044388	ZNF557
hsa-let-7d-5p	NM_031220	PITPNM3

hsa-let-7d-5p	NM_022918	TMEM135
hsa-let-7d-5p	NM_001168724	TMEM135
hsa-let-7d-5p	NR_033149	TMEM135
hsa-let-7d-5p	NM_032866	CGNL1
hsa-let-7d-5p	NM_033401	CNTNAP4
hsa-let-7d-5p	NM_017784	OSBPL10
hsa-let-7d-5p	NM_001174060	OSBPL10
hsa-let-7d-5p	NM_001099694	ZNF578
hsa-let-7d-5p	NM_001206670	PLA2G4E
hsa-let-7d-5p	NM_020877	DNAH2
hsa-let-7d-5p	NM_024884	L2HGDH
hsa-let-7d-5p	NR_015409	LOC143188
hsa-let-7d-5p	NM_152336	AGBL1
hsa-let-7d-5p	NM_001040653	ZXDC
hsa-let-7d-5p	NM_001143957	GPR63
hsa-let-7d-5p	NM_199454	PRDM16
hsa-let-7d-5p	NM_022114	PRDM16
hsa-let-7d-5p	NM_207322	C2CD4A
hsa-let-7d-5p	NM_033122	CABS1
hsa-let-7d-5p	NM_024740	ALG9
hsa-let-7d-5p	NM_001077690	ALG9
hsa-let-7d-5p	NM_001077691	ALG9
hsa-let-7d-5p	NM_001077692	ALG9
hsa-let-7d-5p	NM_001034841	ITPRIPL2
hsa-let-7d-5p	NR_028028	ITPRIPL2
hsa-let-7d-5p	NM_032717	AGPAT9
hsa-let-7d-5p	NM_173495	PTCHD1
hsa-let-7d-5p	NM_153366	SVEP1
hsa-let-7d-5p	NM_001080457	LRRC4B
hsa-let-7d-5p	NM_138400	NOM1
hsa-let-7d-5p	NM_001165966	PITPNM3
hsa-let-7d-5p	NM_080876	DUSP19
hsa-let-7d-5p	NM_080876	DUSP19
hsa-let-7d-5p	NM_080876	DUSP19
hsa-let-7d-5p	NM_001142314	DUSP19
hsa-let-7d-5p	NM_001142314	DUSP19
hsa-let-7d-5p	NM_001142314	DUSP19
hsa-let-7d-5p	NM_001105195	FAM123C
hsa-let-7d-5p	NM_001105194	FAM123C
hsa-let-7d-5p	NM_001105193	FAM123C
hsa-let-7d-5p	NM_152698	FAM123C
hsa-let-7d-5p	NM_144613	COX6B2
hsa-let-7d-5p	NM_032237	SGK196
hsa-let-7d-5p	NM_001005210	LRRC55
hsa-let-7d-5p	NM_001197026	PLEKHA8
hsa-let-7d-5p	NM_022834	VWA1
hsa-let-7d-5p	NM_199121	VWA1
hsa-let-7d-5p	NM_023018	NADK
hsa-let-7d-5p	NM_001198993	NADK
hsa-let-7d-5p	NM_001198994	NADK
hsa-let-7d-5p	NM_138994	CNTNAP4
hsa-let-7d-5p	NM_152616	TRIM42
hsa-let-7d-5p	NR_003525	LRRC37A6P
hsa-let-7d-5p	NR_038970	LINC00641

hsa-let-7d-5p	NR_038971	LINC00641
hsa-let-7d-5p	NM_001010891	MTX3
hsa-let-7d-5p	NM_001167741	MTX3
hsa-let-7d-5p	NM_182580	CYB561D1
hsa-let-7d-5p	NM_001134400	CYB561D1
hsa-let-7d-5p	NM_001134402	CYB561D1
hsa-let-7d-5p	NM_001134403	CYB561D1
hsa-let-7d-5p	NM_001134404	CYB561D1
hsa-let-7d-5p	NR_024207	KIAA1875
hsa-let-7d-5p	NM_207454	C17orf102
hsa-let-7d-5p	NR_024497	LOC399744
hsa-let-7d-5p	NM_198514	NHLRC2
hsa-let-7d-5p	NM_207396	RNF207
hsa-let-7d-5p	NM_173827	COX18
hsa-let-7d-5p	NR_027033	MIRLET7BHG
hsa-let-7d-5p	NM_199340	LRRC37A3
hsa-let-7d-5p	NM_199340	LRRC37A3
hsa-let-7d-5p	NM_198580	SLC27A1
hsa-let-7d-5p	NM_173570	ZDHHC23
hsa-let-7d-5p	NM_152778	MFSD8
hsa-let-7d-5p	NM_173654	EOGT
hsa-let-7d-5p	NM_178173	CCDC36
hsa-let-7d-5p	NM_178518	TMEM102
hsa-let-7d-5p	NM_173689	CRB2
hsa-let-7d-5p	NM_173593	B4GALNT3
hsa-let-7d-5p	NR_027410	GOLGA8B
hsa-let-7d-5p	NR_033738	LOC440300
hsa-let-7d-5p	NM_001012659	ARGFX
hsa-let-7d-5p	NM_001006607	LRRC37A2
hsa-let-7d-5p	NR_033834	FLJ31485
hsa-let-7d-5p	NM_001201380	CNTNAP3B
hsa-let-7d-5p	NM_001144989	ZNF814
hsa-let-7d-5p	NR_038343	MAGI2-AS3
hsa-let-7d-5p	NR_028393	TSNAX-DISC1
hsa-let-7d-5p	NR_037928	P2RX5-TAX1BP3
hsa-let-7d-5p	NR_033378	LOC100288428
hsa-let-7d-5p	NM_181701	QSOX2
hsa-let-7d-5p	NM_013389	NPC1L1
hsa-let-7d-5p	NM_001621	AHR
hsa-let-7d-5p	NM_144997	FLCN
hsa-let-7d-5p	NM_001020818	MYADM
hsa-let-7d-5p	NM_001020819	MYADM
hsa-let-7d-5p	NM_001135659	NRXN1
hsa-let-7d-5p	NM_004801	NRXN1
hsa-let-7d-5p	NM_014683	ULK2
hsa-let-7d-5p	NM_177438	DICER1
hsa-let-7d-5p	NM_030621	DICER1
hsa-let-7d-5p	NM_000903	NQO1
hsa-let-7d-5p	NM_001025433	NQO1
hsa-let-7d-5p	NM_001025434	NQO1
hsa-let-7d-5p	NM_003907	EIF2B5
hsa-let-7d-5p	NM_001034173	ALDH1L2
hsa-let-7d-5p	NR_027752	ALDH1L2
hsa-let-7d-5p	NM_002697	POU2F1

hsa-let-7d-5p	NM_001198783	POU2F1
hsa-let-7d-5p	NM_001198786	POU2F1
hsa-let-7d-5p	NR_037163	POU2F1
hsa-let-7d-5p	NM_022817	PER2
hsa-let-7d-5p	NM_001195573	DICER1
hsa-let-7d-5p	NM_005653	TFCP2
hsa-let-7d-5p	NM_022905	TTC23
hsa-let-7d-5p	NM_001040655	TTC23
hsa-let-7d-5p	NM_001040658	TTC23
hsa-let-7d-5p	NM_001204172	MDM4
hsa-let-7d-5p	NM_002011	FGFR4
hsa-let-7d-5p	NM_213647	FGFR4
hsa-let-7d-5p	NM_017617	NOTCH1
hsa-let-7d-5p	NR_002728	KCNQ1OT1
hsa-let-7d-5p	NM_005497	GJC1
hsa-let-7d-5p	NM_001080383	GJC1
hsa-let-7d-5p	NM_022963	FGFR4
hsa-let-7d-5p	NM_000552	VWF
hsa-let-7d-5p	NM_002205	ITGA5
hsa-let-7d-5p	NM_022842	CDCP1
hsa-let-7d-5p	NM_000870	HTR4
hsa-let-7d-5p	NM_001040173	HTR4
hsa-let-7d-5p	NM_003213	TEAD4
hsa-let-7d-5p	NM_201441	TEAD4
hsa-let-7d-5p	NM_201443	TEAD4
hsa-let-7d-5p	NM_002392	MDM2
hsa-let-7d-5p	NM_002393	MDM4
hsa-let-7d-5p	NR_024171	MDM4
hsa-let-7d-5p	NM_001204171	MDM4
hsa-let-7d-5p	NR_003570	FLJ46361
hsa-let-7d-5p	NR_027240	LOC730668
hsa-let-7d-5p	NR_026804	FLJ13197
hsa-let-7d-5p	NM_004628	XPC
hsa-let-7d-5p	NM_001145769	XPC
hsa-let-7d-5p	NM_001031854	ACCSL
hsa-let-7d-5p	NR_034027	LOC338739
hsa-let-7d-5p	NR_036536	SNHG4
hsa-let-7d-5p	NR_003141	SNHG4
hsa-let-7d-5p	NR_027765	SBF1P1
hsa-let-7d-5p	NM_003496	TRRAP
hsa-let-7d-5p	NM_001244580	TRRAP
hsa-miR-106b-5p	NM_178457	ZNF831
hsa-miR-106b-5p	NM_022112	TP53AIP1
hsa-miR-106b-5p	NM_001195195	TP53AIP1
hsa-miR-106b-5p	NM_175056	ZPLD1
hsa-miR-106b-5p	NM_004226	STK17B
hsa-miR-106b-5p	NR_027673	ADARB1
hsa-miR-106b-5p	NM_001105244	PTPRM
hsa-miR-106b-5p	NM_000051	ATM
hsa-miR-106b-5p	NR_027672	ADARB1
hsa-miR-106b-5p	NM_001112	ADARB1
hsa-miR-106b-5p	NM_015833	ADARB1
hsa-miR-106b-5p	NM_015834	ADARB1
hsa-miR-106b-5p	NR_027674	ADARB1

hsa-miR-106b-5p	NM_001160230	ADARB1
hsa-miR-106b-5p	NM_133178	PTPRU
hsa-miR-106b-5p	NM_133177	PTPRU
hsa-miR-106b-5p	NM_005704	PTPRU
hsa-miR-106b-5p	NM_001195001	PTPRU
hsa-miR-106b-5p	NM_014798	PLEKHM1
hsa-miR-106b-5p	NR_027774	PLEKHM1
hsa-miR-106b-5p	NR_027782	PLEKHM1
hsa-miR-106b-5p	NM_004631	LRP8
hsa-miR-106b-5p	NM_001018054	LRP8
hsa-miR-106b-5p	NM_033300	LRP8
hsa-miR-106b-5p	NM_207123	GAB1
hsa-miR-106b-5p	NM_002039	GAB1
hsa-miR-106b-5p	NM_002845	PTPRM
hsa-miR-106b-5p	NM_017522	LRP8
hsa-miR-106b-5p	NM_005027	PIK3R2
hsa-miR-106b-5p	NM_018240	KIRREL
hsa-miR-106b-5p	NM_017757	ZNF407
hsa-miR-106b-5p	NM_001146189	ZNF407
hsa-miR-106b-5p	NM_001226	CASP6
hsa-miR-106b-5p	NM_032992	CASP6
hsa-miR-106b-5p	NM_001007267	PLA2R1
hsa-miR-106b-5p	NM_001195641	PLA2R1
hsa-miR-106b-5p	NM_001128608	MAPKBP1
hsa-miR-106b-5p	NM_001039199	TTPAL
hsa-miR-106b-5p	NM_172207	CAMKK1
hsa-miR-106b-5p	NM_032116	KATNAL1
hsa-miR-106b-5p	NM_001014380	KATNAL1
hsa-miR-106b-5p	NM_052859	RFT1
hsa-miR-106b-5p	NM_024756	MMRN2
hsa-miR-106b-5p	NR_037633	GJA9-MYCBP
hsa-miR-106b-5p	NR_037634	GJA9-MYCBP
hsa-miR-106b-5p	NR_037146	TNFSF12
hsa-miR-106b-5p	NM_003809	TNFSF12
hsa-miR-106b-5p	NM_005204	MAP3K8
hsa-miR-130a-3p	NM_032160	DSEL
hsa-miR-130a-3p	NM_000876	IGF2R
hsa-miR-130a-3p	NM_016491	MRPL37
hsa-miR-130a-3p	NM_001080431	SLC45A4
hsa-miR-130a-3p	NM_015029	POP1
hsa-miR-130a-3p	NM_001145860	POP1
hsa-miR-130a-3p	NM_001145861	POP1
hsa-miR-130a-3p	NR_029498	MIR25
hsa-miR-146a-5p	NM_004775	B4GALT6
hsa-miR-146a-5p	NM_001025242	IRAK1
hsa-miR-146a-5p	NM_001025243	IRAK1
hsa-miR-146a-5p	NM_025259	MSH5
hsa-miR-146a-5p	NM_172165	MSH5
hsa-miR-146a-5p	NM_001296	CCBP2
hsa-miR-146a-5p	NM_019024	HEATR5B
hsa-miR-146a-5p	NM_016267	VGLL1
hsa-miR-146a-5p	NM_020693	DSCAML1
hsa-miR-146a-5p	NM_001083614	EARS2
hsa-miR-146a-5p	NR_003501	EARS2

hsa-miR-146a-5p	NM_153212	GJB4
hsa-miR-146a-5p	NM_030630	HID1
hsa-miR-146a-5p	NM_001001694	IL17REL
hsa-miR-146a-5p	NM_003954	MAP3K14
hsa-miR-146a-5p	NR_037846	MSH5-SAPCD1
hsa-miR-146a-5p	NM_014983	HMGXB3
hsa-miR-146a-5p	NM_001037131	AGAP1
hsa-miR-146a-5p	NM_014914	AGAP1
hsa-miR-15a-5p	NM_001005405	KRTAP5-11
hsa-miR-15a-5p	NM_001012709	KRTAP5-4
hsa-miR-15a-5p	NM_001012710	KRTAP5-10
hsa-miR-15a-5p	NM_001012416	KRTAP5-6
hsa-miR-15a-5p	NM_021046	KRTAP5-8
hsa-miR-15a-5p	NM_001001480	KRTAP5-5
hsa-miR-15a-5p	NM_001012503	KRTAP5-7
hsa-miR-15a-5p	NM_001012708	KRTAP5-3
hsa-miR-15a-5p	NM_001005922	KRTAP5-1
hsa-miR-15a-5p	NM_152833	C9orf69
hsa-miR-15a-5p	NM_030626	LRRC27
hsa-miR-15a-5p	NM_001143757	LRRC27
hsa-miR-15a-5p	NM_032173	ZNRF3
hsa-miR-15a-5p	NM_001206998	ZNRF3
hsa-miR-15a-5p	NM_015680	CNPPD1
hsa-miR-15a-5p	NM_001032221	STXBP1
hsa-miR-15a-5p	NM_003165	STXBP1
hsa-miR-15a-5p	NM_005877	SF3A1
hsa-miR-15a-5p	NM_001005409	SF3A1
hsa-miR-15a-5p	NM_002045	GAP43
hsa-miR-15a-5p	NM_001130064	GAP43
hsa-miR-15a-5p	NM_005553	KRTAP5-9
hsa-miR-15a-5p	NM_000833	GRIN2A
hsa-miR-15a-5p	NM_001185077	ARHGDIA
hsa-miR-15a-5p	NM_001185078	ARHGDIA
hsa-miR-15a-5p	NM_005919	MEF2BNB-MEF2B
hsa-miR-15a-5p	NR_027307	MEF2BNB-MEF2B
hsa-miR-15a-5p	NR_027308	MEF2BNB-MEF2B
hsa-miR-15a-5p	NM_002590	PCDH8
hsa-miR-15a-5p	NM_032949	PCDH8
hsa-miR-15a-5p	NM_006826	YWHAQ
hsa-miR-15a-5p	NM_005501	ITGA3
hsa-miR-15a-5p	NM_002204	ITGA3
hsa-miR-15a-5p	NM_017736	THUMPD1
hsa-miR-15a-5p	NM_015937	PIGT
hsa-miR-15a-5p	NM_001184728	PIGT
hsa-miR-15a-5p	NM_001184729	PIGT
hsa-miR-15a-5p	NM_001184730	PIGT
hsa-miR-15a-5p	NM_024490	ATP10A
hsa-miR-15a-5p	NM_018199	EXD2
hsa-miR-15a-5p	NM_017982	SUSD4
hsa-miR-15a-5p	NM_001193360	EXD2
hsa-miR-15a-5p	NM_001193361	EXD2
hsa-miR-15a-5p	NM_001193362	EXD2
hsa-miR-15a-5p	NM_001193363	EXD2
hsa-miR-15a-5p	NR_034164	EXD2

hsa-miR-15a-5p	NR_034165	EXD2
hsa-miR-15a-5p	NM_015531	C2CD3
hsa-miR-15a-5p	NR_003266	LOC220729
hsa-miR-15a-5p	NM_080552	SLC32A1
hsa-miR-15a-5p	NM_024575	TNFAIP8L2
hsa-miR-15a-5p	NM_024980	GPR157
hsa-miR-15a-5p	NM_138697	TAS1R1
hsa-miR-15a-5p	NM_022117	TSPYL2
hsa-miR-15a-5p	NM_052902	STK11IP
hsa-miR-15a-5p	NM_031935	HMCN1
hsa-miR-15a-5p	NM_130759	GIMAP1
hsa-miR-15a-5p	NM_001142935	MXD3
hsa-miR-15a-5p	NM_152228	TAS1R3
hsa-miR-15a-5p	NM_173569	UBN2
hsa-miR-15a-5p	NR_029663	MIR15B
hsa-miR-15a-5p	NM_182632	SLC6A18
hsa-miR-15a-5p	NM_001145026	PTPRQ
hsa-miR-15a-5p	NM_173567	EPHX4
hsa-miR-15a-5p	NM_001145785	MEF2B
hsa-miR-15a-5p	NM_014324	AMACR
hsa-miR-15a-5p	NM_203382	AMACR
hsa-miR-15a-5p	NM_001167595	AMACR
hsa-miR-15a-5p	NM_003072	SMARCA4
hsa-miR-15a-5p	NM_001128844	SMARCA4
hsa-miR-15a-5p	NM_001128845	SMARCA4
hsa-miR-15a-5p	NM_001128846	SMARCA4
hsa-miR-15a-5p	NM_001128847	SMARCA4
hsa-miR-15a-5p	NM_001128848	SMARCA4
hsa-miR-15a-5p	NM_001128849	SMARCA4
hsa-miR-15a-5p	NM_002998	SDC2
hsa-miR-15a-5p	NM_004168	SDHA
hsa-miR-15a-5p	NM_000255	MUT
hsa-miR-15a-5p	NM_003920	TIMELESS
hsa-miR-15a-5p	NM_015225	PRUNE2
hsa-miR-15a-5p	NM_033637	BTRC
hsa-miR-15a-5p	NM_152594	SPRED1
hsa-miR-15a-5p	NM_004613	TGM2
hsa-miR-15a-5p	NM_198951	TGM2
hsa-miR-15a-5p	NM_003939	BTRC
hsa-miR-15a-5p	NR_003264	SDHAP1
hsa-miR-15a-5p	NR_003265	SDHAP2
hsa-miR-194-5p	NM_000862	HSD3B1
hsa-miR-194-5p	NM_005819	STX6
hsa-miR-194-5p	NM_001981	EPS15
hsa-miR-194-5p	NM_001159969	EPS15
hsa-miR-194-5p	NM_003128	SPTBN1
hsa-miR-194-5p	NM_001127221	CACNA1A
hsa-miR-194-5p	NM_012194	KIAA1549L
hsa-miR-194-5p	NM_012090	MACF1
hsa-miR-194-5p	NM_002758	MAP2K6
hsa-miR-194-5p	NM_001077203	SENP7
hsa-miR-194-5p	NM_016376	ANKFY1
hsa-miR-194-5p	NM_007041	ATE1
hsa-miR-194-5p	NM_001001976	ATE1

hsa-miR-194-5p	NM_015442	CNOT10
hsa-miR-194-5p	NR_023938	C14orf132
hsa-miR-194-5p	NM_001002915	IGFL2
hsa-miR-194-5p	NM_001135113	IGFL2
hsa-miR-194-5p	NM_022164	TINAGL1
hsa-miR-194-5p	NM_032840	SPRYD3
hsa-miR-194-5p	NM_001204414	TINAGL1
hsa-miR-194-5p	NM_001135748	NEIL2
hsa-miR-194-5p	NR_029711	MIR194-1
hsa-miR-194-5p	NM_024814	CBLL1
hsa-miR-194-5p	NR_024199	CBLL1
hsa-miR-194-5p	NM_017666	ZNF280C
hsa-miR-194-5p	NR_002816	THSD1P1

Abbreviation: The gene symbol is presented as in NCBI at http://www.ncbi.nlm.nih.gov.

Supplementary Table S4. GO terms significantly over-represented among the deregulated miRNA targets.

Term	Description	P-value
biological process		
GO:0051128	regulation of cellular component organization	1.30E-04
GO:0030100	regulation of endocytosis	1.20E-03
GO:0002755	MyD88-dependent toll-like receptor signaling pathway	1.52E-03
GO:0035556	intracellular signal transduction	2.94E-03
GO:0060627	regulation of vesicle-mediated transport	4.26E-03
GO:0007509	mesoderm migration involved in gastrulation	1.50E-02
GO:0001909	leukocyte mediated cytotoxicity	1.89E-02
GO:0007399	nervous system development	2.03E-02
GO:0048583	regulation of response to stimulus	3.52E-02
GO:0009966	regulation of signal transduction	3.77E-02
cellular component		
GO:0048770	pigment granule	4.80E-04
GO:0030135	coated vesicle	3.90E-03
GO:0031988	membrane-bounded vesicle	5.20E-03
GO:0043205	fibril	1.84E-02
GO:0030136	clathrin-coated vesicle	2.08E-02
GO:0031410	cytoplasmic vesicle	3.94E-02
GO:0016023	cytoplasmic membrane-bounded vesicle	4.35E-02
GO:0030424	axon	4.61E-02
molecular function		
GO:0004000	adenosine deaminase activity	1.98E-02
GO:0019239	deaminase activity	1.98E-02
GO:0019899	enzyme binding	2.79E-02

Supplementary Table S5. KEGG pathway analysis results.

Pathway name	Hits	P value	Q value	Gene symbol
Axon guidance	8	1.11E-3	1.10E-2	LRRC37B;LRRC37A;LRRC4B;LRRC55;LRRC37A6P; LRRC37A2;KIRREL;STK11IP
Neuroactive ligand-receptor interaction	9	2.87E-3	2.36E-2	LRRC37A;F2RL2;GNRHR2;LOC399744;LRRC37A2; P2RX5-TAX1BP3;HTR4;LOC730668; GRIN2A
MAPK signaling pathway	10	3.66E-4	4.06E-3	CACNA1A;CACNA1H;DUSP7;DUSP9;DUSP16; PLA2G4E;PRDM16;FGFR4;MAP3K8; MAP3K14
Calcium signaling pathway	6	1.08E-3	1.10E-2	CACNA1A; CACNA1H;P2RX5-TAX1BP3;HTR4; LOC730668; GRIN2A
Neurotrophin signaling pathway	6	1.21E-3	1.12E-2	ZNF274;GAB1;ARHGDIA;YWHAQ; PIK3R2; IRAK1
Apoptosis	6	1.52E-3	1.33E-2	CASP6; IRAK1; ATM; MAP3K14; MAPKBP1; PIK3R2

Abbreviation: Hits, number of potential target genes in the pathway. The gene symbol is presented as in NCBI at http://www.ncbi.nlm.nih.gov.

Supplementary Table S6.Mean Cq value of mRNAs in RT-PCR.

Samples	GAPDH	IRAK1	CASP6	MAPKBP1	MAP2K6
c1	16.53	20.43	21.44	21.17	23.41
c2	17.22	21.2	22.5	21.22	23.57
c3	16.96	19.93	21.77	21.17	24.51
c4	16.59	20.05	21	21.45	22.27
c5	16.32	20.81	21.57	20.09	23.92
c6	16.29	21.06	22.79	20.51	22.65
c7	17.44	21.12	23.5	21.56	23.49
c8	16.96	22.04	22.56	20.88	24.05
c9	17.31	21.19	24.05	23.01	24.58
c10	16.72	20.83	21.88	21.49	23.58
c11	16.66	20.04	21.69	20.43	24.02
c12	17.21	21.58	23.09	22.11	22.29
c13	17.51	21.97	22.69	22.69	23.59
c14	17.22	22.54	22.81	22.75	23.72
c15	16.48	21.12	21.47	21.62	23.29
c16	16.54	20.69	20.84	21.11	22.18
c17	17.33	22	23.51	21.26	24.19
c18	17.47	21.43	24	22.31	23.64
c19	16.57	22.01	23.16	21.98	23.29
c20	16.73	21.1	23.03	21.84	22.83
c21	16.48	20.93	21.99	20.59	21.49
c22	17.17	21.17	23.17	21.89	24.3
c23	17.22	23.04	22.5	21.97	21.85
c24	16.54	21.2	21.55	21.47	22.93
c25	16.73	21.82	21.93	20.98	24
c26	17.37	21.02	23.57	22.36	22.49
c27	16.81	20.72	22.77	21.72	24.04
c28	16.76	20.42	21.21	21.74	23.4
c29	16.64	19.72	24.04	21.07	23.38
c30	17.27	20.45	23.17	22.38	23.59
c31	17.19	21.34	23.23	21.72	22.65
c32	16.52	21.01	22.1	21.71	22.92
ep1	16.49	21.65	23.44	22.12	22.16
ep2	17.11	22.04	24.31	22.7	22.61
ep3	16.72	21.61	23.93	22.61	21.12
ep4	16.39	20.95	21.88	21.95	21.83
ep5	16.7	21.73	22.17	22.79	21.95
ep6	16.82	22.37	22.9	21.47	23.3
ep7	17.3	23.27	23.59	20.97	23.19
ep8	17.35	23.12	24.51	23.06	23.02
ep9	16.56	22.13	24.04	22.06	21.21
ep10	16.69	21.92	24.24	21.79	23.82
ep11	17.22	22.06	21.78	21.62	23.38
ep12	17.4	22.22	24.5	22.74	23.5
ep13	17.17	23	21.47	23.47	23.89
ep14	16.62	21.61	23.55	22.58	21.2
ep15	16.79	22.19	22.61	21.48	22.59
ep16	17.47	21.83	22.7	23.17	22.92
ep17	16.53	21.4	22.83	22.67	22.68
ep18	17.27	21.73	23.9	21.37	22
ep19	17.21	22.48	23.69	22.83	22.56

ep20	16.77	22.05	24.56	22.96	21.72
ep21	16.7	21.92	24.42	22.06	23.62
ep22	17.26	23.58	23.86	24.61	22.54
ep23	17.37	22.21	24.63	23.95	23.3
ep24	16.88	22.1	24.11	23.1	21.62
ep25	16.79	21.79	23.44	23.26	21.61
ep26	16.56	22.02	22.67	22.9	23.2
ep27	16.39	21.02	21.55	21.99	21.6
ep28	17.33	23.37	21.79	22.69	23.26
ep29	17.26	22.1	22.03	23.61	22.99
ep30	16.54	22.01	21.68	21.61	21.57
ep31	17.03	22.52	23.57	24.04	21.63
ep32	16.93	21.97	24.34	22.5	22.45
ep33	17.07	22.87	24.55	23.59	22.16
ep34	16.36	21.58	23.82	22.42	22.34
